# Matrix structure and microenvironment dynamics correlate with chemotherapy response in ovarian cancer

**DOI:** 10.1016/j.isci.2026.114858

**Published:** 2026-02-09

**Authors:** Florian Laforêts, Panoraia Kotantaki, Samar Elorbany, Joseph Hartlebury, Joash D. Joy, Beatrice Malacrida, Rachel C. Bryan-Ravenscroft, Chiara Berlato, Erica Di Federico, John F. Marshall, Ranjit Manchanda, Wolfgang Jarolimek, Lara Perryman, Eleni Maniati, Frances R. Balkwill

**Affiliations:** 1 Barts Cancer Institute, Queen Mary University of London, Charterhouse Square, London ECM1 6BQ, UK; 2 School of Engineering and Materials Science, Faculty of Science and Engineering, Queen Mary University of London, Mile End Road, London E1 4NS, UK; 3 Wolfson Institute of Population Health, Queen Mary University of London, Charterhouse Square, London ECM1 6BQ, UK; 4 Department of Gynaecological Oncology, Barts Health NHS Trust, Royal London Hospital, London E1 1BB, UK; 5 Department of Health Services Research and Policy, London School of Hygiene & Tropical Medicine, London WC1H 9SH, UK; 6 Syntara Limited, Unit 2, 20A Rodborough Road, Locked Bag 5015, Frenchs Forest, NSW 2086, Australia

**Keywords:** microenvironment, biological sciences, sequence analysis, cancer

## Abstract

Elevated extracellular matrix (ECM) associates with chemo-resistance and poor prognosis in cancer. Modifying the ECM could enhance response to chemotherapy. We measured chemotherapy-induced changes in the tumor microenvironment (TME) of two high-grade serous ovarian cancer (HGSOC) mouse models with different chemo-sensitivity. Carboplatin/paclitaxel treatment triggered dynamic transcriptional changes in immune and ECM-related pathways, and analyses of the ECM structure revealed modifications in the chemo-sensitive tumors. These changes, observed over twenty days post-chemotherapy, had relevance to HGSOC patient responses. Integrating transcriptomics with ECM structure metrics, we identified ECM targets, including lysyl oxidase (LOX), that might enhance effects of chemotherapy. Given alone or with chemotherapy, a pan-LOX inhibitor, PXS-5505, modulated fibroblast and immune cell distribution and decreased tumor stiffness in the less-responsive mouse model. Moreover, treatment with PXS-5505 before chemotherapy enhanced the therapeutic response. We conclude that pretreatment with ECM targeting agents may improve chemotherapy efficacy by altering ECM structure and immune responses.

## Highlights

• Dynamic ECM and immune transcriptional changes were related to chemotherapy response
• Carboplatin/paclitaxel treatment modified ECM protein structure in sensitive tumors
• Lysyl oxidase inhibitor PXS-5505 changed ECM, fibroblast, and immune cell distribution
• PXS-5505 treatment before chemotherapy improved response in HGSOC tumors

## Graphical abstract

**Figure:**
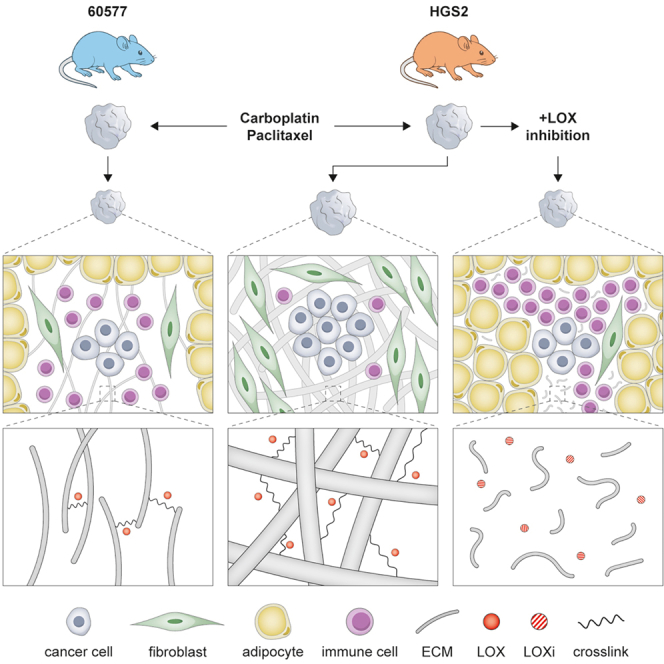

## Introduction

High extracellular matrix (ECM) remodeling (fibrosis) and fibroblast/wound healing transcriptional signatures predict a poor response to therapy and an unfavorable prognosis in many human cancers.^1^^,^^2^^,^^3^^,^^4^ For instance, our previously published multi-level deconstruction of human omental metastases of high-grade serous ovarian cancer (HGSOC) revealed a pattern of ECM molecules, the matrix index, that is associated with a poor prognosis not only in this malignancy but also in twelve other common cancer types.^5^

The majority of HGSOC patients present with advanced disease that has spread throughout the peritoneum and omentum.^6^ They are generally treated with surgery and chemotherapy, often with three cycles of carboplatin/paclitaxel neoadjuvant chemotherapy (NACT), followed by debulking surgery and further three cycles of chemotherapy.^7^

We previously reported that NACT induces anti-tumor immune activity in the human tumor microenvironment (TME), with effects on T cells, B cells, and macrophages.^8^^,^^9^^,^^10^^,^^11^^,^^12^ However, these effects on innate and adaptive immune cells are not usually sufficient to induce long-lasting host anti-tumor activity. Most patients with HGSOC relapse within two years, with increasingly resistant disease.^7^

In this paper, to further investigate the effects of chemotherapy on the TME, we used two of our previously published syngeneic transplantable orthotopic mouse HGSOC models that replicate the human omental TME in terms of ECM composition and immune cells.^13^ The two intra-peritoneal mouse models varied significantly in their response to chemotherapy, with the 60577 model representing a highly chemo-responsive tumor and HGS2 representing a model with marginal response.^13^ Differences between the transcriptomes of untreated 60577 and HGS2 mouse omental tumors predicted response to chemotherapy in an HGSOC patient dataset and were significantly associated with ECM organization, cell adhesion, collagen catabolism, and organization pathways.^13^ The weakly chemo-sensitive HGS2 model had higher levels of ECM proteins, increased numbers of fibroblasts, and a greater matrix index than the chemo-sensitive 60577 model.^13^

Taken together, these results led us to propose that altering the ECM composition and structure could enhance the effects of chemotherapy in a marginally responsive tumor.

To investigate this hypothesis, we conducted transcriptomic, spatial, and structural analyses of the ECM in established omental tumors from our chemo-responsive model, 60577, after three cycles of carboplatin/paclitaxel chemotherapy. We related our results to patient samples and tested whether manipulating the ECM could enhance response in a weakly chemo-sensitive model, HGS2.

## Results

### Chemotherapy induces dynamic ECM and immune changes at the transcriptomic level in chemo-sensitive 60577 HGSOC murine tumors

**Figure 1:**
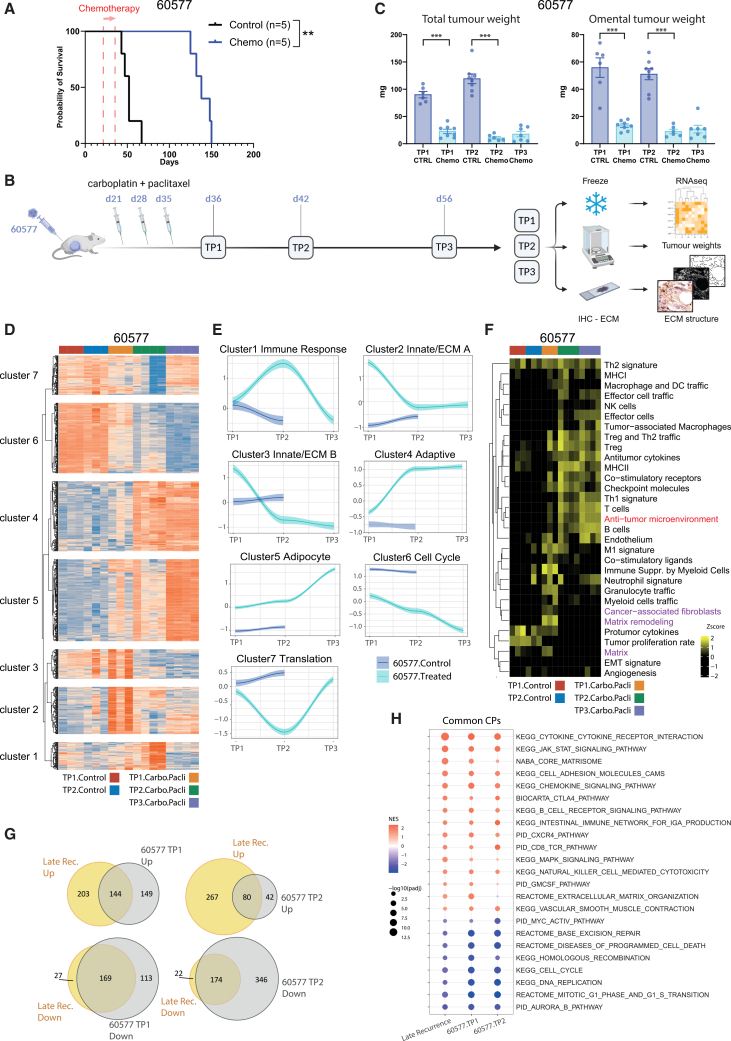
Response to chemotherapy was associated with ECM and immune response pathway alterations in 60577 mouse HGSOC model (A) Response of 60577 tumor-bearing mice to three doses of a carboplatin and paclitaxel combination (chemo, blue) compared with the control group (black). Kaplan-Meier survival curve is shown, with median survival times for control and treated 60577 mice being 52 and 138 days, respectively. The log-rank *p* value is depicted next to the survival curve, ∗∗, *p* < 0.01. The start of the treatment is indicated by the red arrow. Numbers of mice enrolled in each arm are shown in parentheses. (B) Scheme of the animal study designed to assess the effect of carboplatin and paclitaxel on 60577 tumors at different time points. Carboplatin (20 mg/kg) and paclitaxel (10 mg/kg) were administered i.p. once a week for three weeks starting 3 weeks post tumor cell injection. Mice were culled one day (TP1), one week (TP2), and three weeks (TP3) after the last dose of chemotherapy; their tumors were resected, frozen or formalin-fixed, and paraffin embedded after being weighted. RNA was extracted from the frozen tumors and subjected to RNA-seq analysis. FFPE sections were stained for ECM markers to assess ECM structure and texture. (C) Total and omental tumor weights from animals described in (B). Each dot represents a tumor from an individual mouse, and data are represented as the mean ± SEM. Blue and light blue colors show tumor weight of the control (ctrl) and chemotherapy-treated (chemo) mice, respectively. *p* values for Mann-Whitney test are depicted on the bar plots for comparisons between the control and treated mice. No significant difference between treated and control mice was detected at TP1, TP2, and TP3 (Kruskal-Wallis test). *p* < 0.001. (D) Heatmap illustrating log_2_RPKM gene expression for genes with a significant change between 60577 treated vs. control (TP1 and/or TP2) and a significant change between at least one of the three treated time point contrasts (TP1 vs. TP2, TP2 vs. TP3, and TP1 vs. TP3) (*p*_*adj*_ <0.05 and log_2_FC > |1|). Heatmap was clustered using *K*-means clustering into 7 clusters. (E) Graphical illustration of the patterns of gene expression across the 7 clusters. Trendlines for the expression of each cluster were plotted using scaled gene expression and the median value of replicate mice. Graphs were plotted on ggplot with trendline, using the loess smoothing method. (F) Heatmap illustrating GSVA enrichment scores of Bagaev et al.’s^14^ functional gene expression signatures in 60577 control and treated tumors. The anti-TME signature (red) was calculated using the M1 signature, Th1 signature, and anti-tumor cytokines signature genes as well as the B cell signature genes. ECM-related signature is highlighted in fuchsia. Anti-TME, anti- tumor microenvironment. (G) Venn diagrams illustrating the overlap of significantly enriched canonical pathways in the Adzibolosu et al.’s human HGSOC dataset^15^ of late recurrence post-treatment vs. pre-treatment samples and canonical pathways significantly changing in 60577 treated vs. control at TP1 and TP2, *p*_*adj*_ ≤ 0.05. (H) Enrichment dot plot illustrating some of the canonical pathways common to both the human and mouse data.

We first studied the chemo-sensitive 60577 syngeneic peritoneal model of HGSOC, as mentioned above.^13^ The response of established tumors to three doses of carboplatin/paclitaxel (chemo) was confirmed (Figure 1A) with the treatment giving a 12-week survival advantage. To replicate the NACT given to patients, we administered three doses of carboplatin/paclitaxel (chemo) to mice with established disease and collected tumors 24 h (TP1) and one week (TP2) after treatment (Figure 1B). Treated tumors were also obtained three weeks after chemo (TP3), but there were insufficient mice alive in the control group. There was a significant reduction in tumor burden at TP1 and TP2, which was maintained at TP3 (Figure 1C), in agreement with the experiment shown in Figure 1A.

We, therefore, carried out transcriptomic analyses of the control and treated 60577 omental tumors at TP1, TP2, and TP3. Hierarchical cluster analysis of the expressed genes showed that the treated 60577 tumors clustered separately from controls (Figure S1A). We compiled all genes with a significant differential expression between the control and treated tumors. *K*-means clustering identified seven differential expression groups (Figure 1D and Table S1A). We plotted the patterns of gene expression in these clusters (Figure 1E). ECM organization, integrin-mediated signaling, and innate immunity pathways transiently increased at TP1, but decreased at TP2, a change that was sustained at TP3 (clusters 2 and 3). As innate immune pathways decreased, adaptive immune response signaling increased and was sustained as shown in cluster 4. Cell division and cell cycle pathways were reduced in treated tumors (cluster 6), whereas lipid and adipocyte-related pathways increased with the tumor response (cluster 5) (Figures 1E and S1B; Table S1B).

In summary, RNA-seq analysis showed dynamic transcriptional responses twenty days post-treatment, as the 60577 omental tumors responded significantly to chemo. The changes predominantly related to non-malignant cells in the TME. Initial ECM and innate immune gene expression declined as adaptive immune response gene expression increased. This was accompanied by a decrease in pathways associated with cell proliferation and an increase in lipid synthesis gene expression in this adipocyte-rich TME.

In order to understand the relevance of these transcriptomic changes to human cancers, we referred to functional gene expression signatures defined in a pan-cancer human TME transcriptome analysis of 10,000 human tumors.^14^ These pan-cancer signatures captured the stromal and immune compartment of the TME, as well as anti-tumor and tumor-promoting processes. Selecting the mouse orthologues of the human genes in the signatures, we again found dynamic changes in ECM molecules, matrix remodeling, and cancer-associated fibroblast (CAF) pathways, along with TME cell signatures during the three weeks after chemo (Figure 1F and Table S2) that were sustained at TP3. In line with the analysis in Figure 1D, we observed a transient increase in matrix remodeling and innate immune signatures at TP1 in treated tumors, including a transient increase in the signatures of immature myeloid cells and CAFs, suggesting these cell types are part of the remodeling process in responsive tumors. This initial increase in innate processes was followed by an increase in adaptive immune signatures, which became evident at TP2 and were sustained at TP3, including genes involved in Treg and Th2 cell trafficking. These data show that TME gene expression in the 60577 mouse tumors is relevant to human TME transcriptional pathways.

Of particular interest were changes in a “anti-tumor microenvironment” signature enrichment score (red text Figure 1F). The signature is adapted from a compilation of the M1 signature, Th1 signature, and anti-tumor cytokines, as described by Bagaev et al.^14^ We added B cell gene expression pathways to this signature as they relate to good prognosis in ovarian cancer.^16^^,^^17^^,^^18^^,^^19^ Expression levels of our anti-TME score increased after chemotherapy and were sustained (Figure 1F).

We conclude that the anti-tumor immune and ECM responses were induced by chemotherapy. To further relate our findings in the mouse model to patient data, we analyzed a transcriptomic dataset of paired samples from twenty-four HGSOC patients pre- and post- carboplatin/paclitaxel NACT.^15^ This publication reported significant immune changes in post-treatment samples from patients with late disease recurrence compared to those who relapsed early (i.e., chemo-sensitive versus chemo-resistant tumors).^15^ Using mouse orthologues and comparing the changes in treated versus control 60577 tumors with the post versus pre-chemo late recurrence patient samples (i.e., mouse versus human “responders”), we found significant overlap in up- and down-regulated pathways between mouse and human responding tumors (hypergeometric test, *p* < 0.0001, Figure 1G). Again, ECM and immune pathways were significantly related to response in both mouse and human HGSOC tumors (Figure 1H and Table S3).

In summary, the dynamic and sustained changes in ECM and immune pathways observed after chemotherapy in 60577 tumors were relevant to transcriptional pathways in human TMEs. They also reflected changes seen in NACT-treated HGSOC patients.^15^

### Structural alteration of the ECM is a marker of response to chemotherapy in 60577 tumors

**Figure 2:**
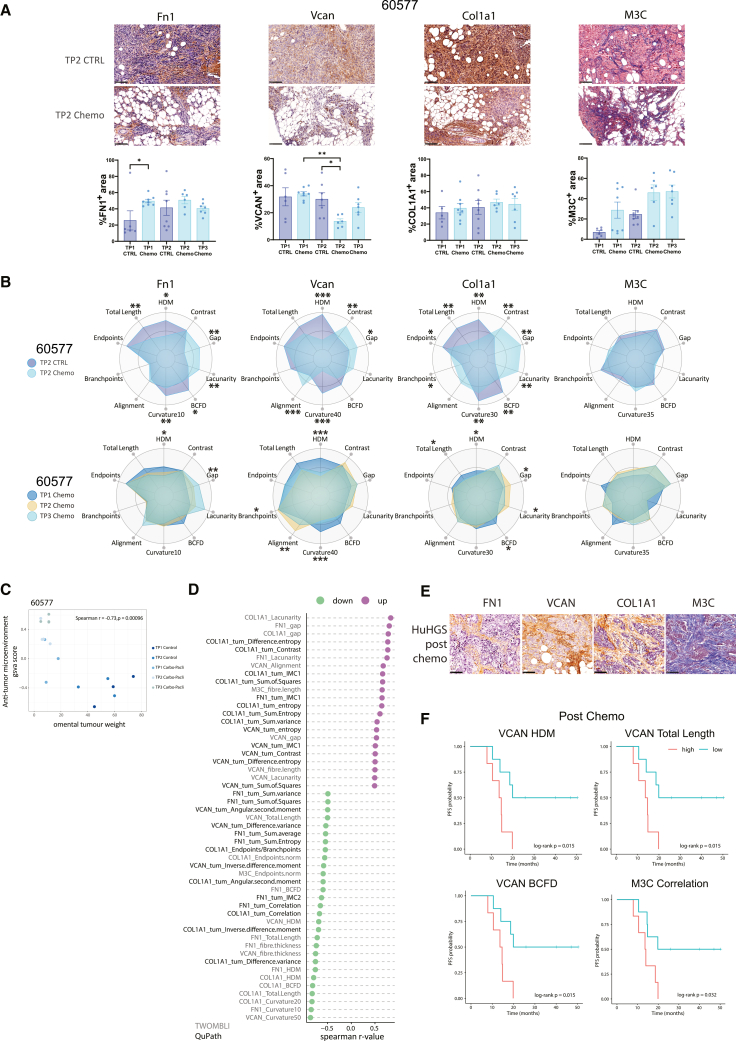
Structural alteration of the ECM is a marker of response to chemotherapy in 60577 tumors (A) Representative immunohistochemistry (IHC) images for fibronectin (FN1), versican (VCAN), collagen 1A1 (COL1A1), and Masson’s Trichrome (M3C) histochemistry of tumors taken at TP2, and the corresponding quantifications of staining in 60577 tumors (blue, control mice; light blue, mice treated with carboplatin and paclitaxel) at TP1, TP2, and TP3. Data are represented as the mean ± SEM; each dot represents one mouse; scale bars correspond to 100 μm. The Mann-Whitney test was used to determine statistical significance between control and treated groups for each individual time point, ∗, *p* < 0.05; ∗∗, *p* < 0.01. The difference between treated groups at time points 1, 2 and 3 (TP1, TP2 and TP3, respectively) was determined with the Kruskal-Wallis test’s multiple comparisons. (B) Matrix patterns for FN1, VCAN, COL1A1, and Masson’s Trichrome (M3C) for 60577 tumors at TP2, assessed by TWOMBLI and Haralick feature analysis and visualized as radar plots. A selection of TWOMBLI metrics along with Haralick contrast is depicted. The average of structural and textural metrics was calculated for the samples analyzed in (A), and the minimum and maximum ranges for each metric were computed across all categories. Values were normalized and scaled to the maximum range. Top panel shows the ECM pattern for control and treated TP2 tumors in dark and light blue, respectively. Bottom panel shows ECM patterns for treated TP1, TP2, and TP3 tumors in blue, beige, and cyan, respectively. HDM, high density matrix; Gap, mean gap area; BCFD, box-counting fractal dimension; Curvature10 to 40, curvature metric was assessed at windows of 10–40 pixels; Branchpoints, normalized branchpoints; Endpoints: normalized endpoints; Fiber Length, average fiber length; ∗, *p* ≤ 0.05; ∗∗ *p* ≤ 0.01, ∗∗∗, *p* ≤ 0.001, Mann-Whitney test. (C) Scatterplot for the correlation of anti-tumor microenvironment (anti-TME) scores with omental tumor weights in 60577. Each dot represents an individual mouse. Points corresponding to different experimental groups are illustrated in different colors. (D) Cleveland plot showing Spearman’s correlations for TWOMBLI and QuPath IHC image analysis metrics that correlated significantly with the anti-TME scores in 60577 (Spearman’s *p* ≤ 0.05 and r ≥ |0.5|). (E) Representative IHC images for FN1, VCAN, COL1A1, and Masson’s Trichrome staining (M3C) in human HGSOC omental biopsies. Scale bars, 100 μm. (F) Kaplan-Meier plots of progression-free survival on post-NACT samples; high and low groups were determined by median values; *n*_high_ = 7; *n*_low_ = 8.

To measure effects of chemotherapy on ECM proteins, we performed IHC on omental tumors obtained from the experiment shown in Figures 1B and 1C, measuring protein levels of three ECM molecules we previously identified as having prognostic significance in patient samples: fibronectin (FN1), versican (VCAN), collagen 1A1 (COL1A1),^5^ and fibrillar collagens. There was a transient reduction in versican staining at TP2 in 60577 tumors but no consistent changes in the levels of the other ECM proteins in the control or treated tumors (Figure 2A). However, TWOMBLI^20^ (structural) and QuPath (Haralick texture features)^21^ analyses revealed significant differences in the structure of FN1, VCAN, and COL1A1 between the control and treated 60577 tumors. Chemo caused a significant reduction in high density matrix (HDM) and curvature with significant increases in gap for the three ECM molecules at TP2. Haralick contrast and lacunarity increased, whereas box-counting fractal dimension (BCFD) and total length decreased for two of the three ECM molecules. These ECM alterations were dynamic and varied between the different time points, with some sustaining at TP3, for instance Col1A1 (Figures 2B and S2A). Fibrillar collagens, as assessed by Masson’s Trichrome (M3C) staining, were mostly unaffected by chemotherapy (Figure 2B).

### Structural ECM features associate with anti-TME transcriptomic signatures

We next integrated these protein metrics with our transcriptomic data. We found a significant negative correlation between 60577 omental tumor weight and the anti-TME score described above (Figure 2C),^14^ reinforcing the link between this signature and response to chemotherapy.

We measured the correlation of all the ECM metrics in 60577 treated and control tumors across all time points with the anti-TME score; those that significantly correlated with the anti-TME score are displayed in a Cleveland plot (Figure 2D). This shows the quantitative structural and textural ECM features that correlate positively (purple) and negatively (green) with the anti-TME score. This gives a picture of decreasing HDM, total length, curvature, fiber thickness, and increases in gap and lacunarity with the anti-TME score, supporting our hypothesis that ECM structure is involved in the response to chemotherapy.

We next assessed if structural changes in ECM proteins were associated with progression-free survival (PFS) in HGSOC patients after NACT. We conducted the same IHC and histochemistry analysis in 15 HGSOC samples collected at interval debulking surgery 3–4 weeks after the third dose of NACT (Figure 2E). We looked for correlations between the ECM structure and texture metrics with PFS. Several metrics correlated with PFS in HGSOC patients, including VCAN total length and VCAN BCFD that were also associated with response in the 60577 tumors (Figure 2D). In the patients, M3C correlation was also associated with PFS (Figures 2F and S2B).

Our results so far show that three doses of chemo triggered dynamic ECM changes in the TME in a chemo-sensitive mouse HGSOC model. The ECM in chemo-treated 60577 tumors was less compact, with increased gaps between fibers. Bioinformatic analysis showed the relevance of these changes to HGSOC patient biopsies, and some ECM structural changes induced by chemo in the mouse tumors were associated with PFS in HGSOC patients.

### Chemotherapy fails to induce transcriptomic and ECM changes in a partially responsive murine HGSOC model, HGS2

**Figure 3:**
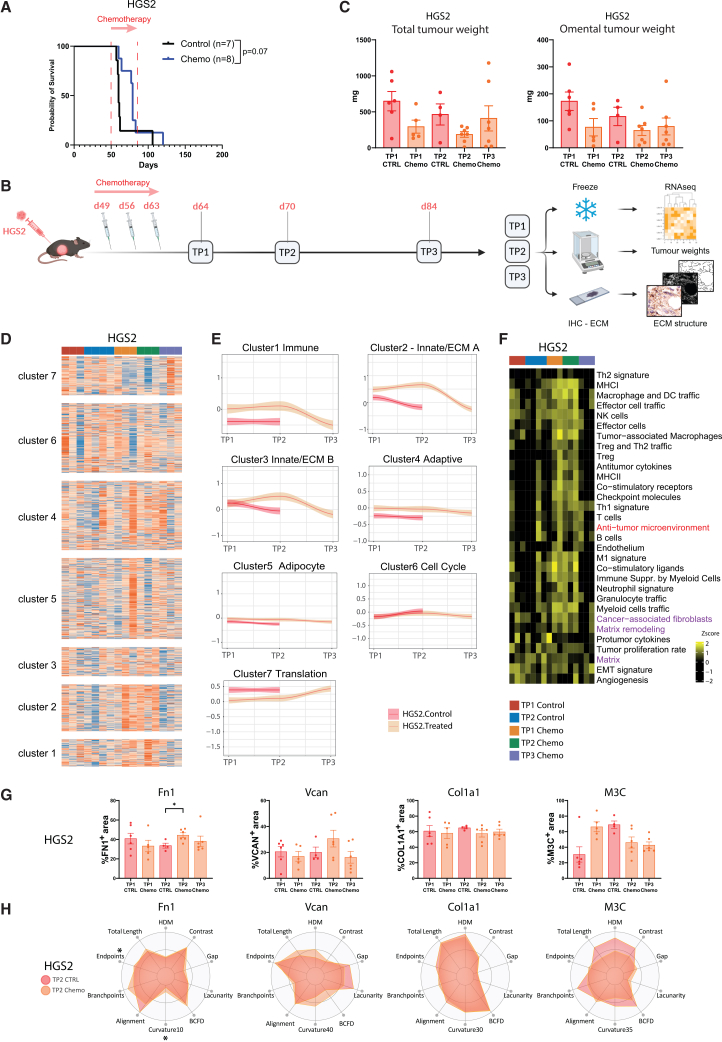
Chemotherapy failed to induce sustained transcriptomic and ECM changes in the HGSOC mouse model HGS2 (A) Responses of HGS2 tumor-bearing mice to six doses of a carboplatin and paclitaxel combination (chemo, blue) compared to the control group (black). Kaplan-Meier survival curves are shown, with median survival for control and treated HGS2 being 60 and 79 days, respectively. The log-rank *p* value is depicted next to the survival curves. The start of the treatment is indicated by the red arrow. Numbers of mice enrolled in each arm are shown in parentheses. (B) Scheme of animal study designed to assess the effect of carboplatin and paclitaxel on HGS2 orthotopic mouse model tumors, at time point. Carboplatin (20 mg/kg) and paclitaxel (10 mg/kg) were administered i.p. once a week for three weeks starting 7 weeks after tumor cell injection. Mice were culled one day (TP1), one week (TP2), and three weeks (TP3) after the last dose of chemotherapy; their tumors were resected, frozen or formalin-fixed, and paraffin embedded after being weighted. RNA was extracted from the frozen tumors and subjected to RNA-seq analysis. FFPE sections were stained for ECM markers to assess ECM structure and texture. (C) Total and omental tumor weights from animals described in (B). Each dot represents a tumor from an individual mouse, with data represented as the mean ± SEM. Dark orange denotes HGS2 control; light orange denotes HGS2 chemo-treated mice, whereas light blue and orange are the respective treated samples from each mouse model colored. *p* values for Mann-Whitney test are depicted on the bar plots for comparisons between the control and treated mice. No significant difference was detected between TP1, TP2, and TP3 (Kruskal-Wallis test). (D) Heatmap illustrating log_2_RPKM gene expression for genes making up the seven clusters identified in 60577 tumors, shown in Figure 1D, in HGS2 tumors. (E) Graphical illustration of the patterns of gene expression across the seven clusters in HGS2 tumors. Trendlines for the expression of each cluster were plotted using scaled gene expression and the median value of replicate mice. Graphs were plotted on ggplot with trendline, using the loess smoothing method. (F) Heatmap illustrating GSVA enrichment scores from Bagaev et al.^14^ functional gene expression signatures in HGS2 tumor. The anti-TME signature was calculated using the M1 signature, Th1 signature, and anti-tumor cytokine signature genes as well as the B cell signature genes. ECM-related signature is highlighted in fuchsia. Anti-TME, anti- tumor microenvironment. (G) Quantifications of fibronectin (FN1), versican (VCAN), and collagen 1A1 (COL1A1) immunohistochemistry and Masson’s Trichrome (M3C) histochemistry staining in HGS2 tumors (red, control mice; orange, mice treated with carboplatin and paclitaxel). Data are represented as the mean ± SEM. Each dot represents one mouse. The Mann-Whitney test was used to determine statistical significance between the control and treated groups for each individual time point. The difference between the treated groups at TP1, TP2, and TP3 was determined with the Kruskal-Wallis test’s multiple comparisons; ∗, *p* < 0.05. (H) Matrix patterns for FN1, VCAN, COL1A1, and M3C for HGS2 tumors at TP2, as they were assessed by TWOMBLI and Haralick feature analysis and visualized in the radar plots. A selection of TWOMBLI metrics along with Haralick contrast is depicted. The average of structural and textural metrics was calculated for the samples analyzed in (B), and the minimum and maximum ranges for each metric were computed across all categories. Values were normalized and scaled to the maximum range. Red and orange show the ECM pattern for control and treated tumors, respectively. HDM, high density matrix; Gap, mean gap area; BCFD, box-counting fractal dimension; Curvature10 to 40, Curvature metric assessed at windows of 10–40 pixels; Branchpoints, normalized branchpoints; Endpoints, normalized endpoints; Fiber Length, average fiber length. ∗, *p* ≤ 0.05; Mann-Whitney test.

Having established that chemotherapy induced dynamic ECM and immune changes in the chemo-sensitive 60577 tumors, we asked if such changes were observed in a less responsive model. Treatment of established HGS2 tumors with six doses of chemo had limited survival benefit (Figure 3A), confirming our previously published results.^13^ We next treated HGS2 tumor-bearing mice with three doses of carboplatin and paclitaxel to resemble NACT (Figure 3B). As with the 60577 model, HGS2 control and chemo-treated tumors were collected at three time points, TP1, TP2, and TP3. There was no HGS2 TP3 control group due to an insufficient number of mice alive. Chemo did not cause a decrease in tumor weights at either time point (Figure 3C).

Next, we conducted bulk RNA-seq in HGS2 control and treated tumors. In this model, chemotherapy-treated tumors did not cluster separately from the controls (Figure S3A), and HGS2 omental tumor weight did not correlate with the anti-TME score (Figure S3B). We looked at expression of the genes that made up the seven clusters described in Figure 1D. These showed no significant differences in HGS2 (Figure 3D) between the control and treated tumors, and plotting the cluster dynamic expression showed little to no variation over time (Figure 3E). We noted changes in HGS2 that overlapped with 60577 tumor results but only if an unadjusted *p* value < 0.01 was used (Figure S3C), though we could not distinguish strong dynamic patterns of alterations between control and treated group that were sustained at TP3 (Figure S3D).

We used the pan-cancer human functional gene signatures identified by Bagaev et al.,^14^ noting a limited number of differences between the control and chemo-treated HGS2 tumors. Importantly, while some TME gene signatures showed weak alterations in treated tumors at TP1 and TP2, these reverted to control levels at TP3 (Figure 3F and Table S2).

We next assessed whether chemotherapy affected the ECM in HGS2 tumors (Figures 3G and S3E). Apart from a transient increase in FN1 at TP2, HGS2 tumors displayed stable levels of ECM molecules. Similarly, the analysis of structural and textural features showed no difference between the control and treated tumors at TP2 for any ECM marker analyzed (Figure 3H). Changes in fibrillar collagen were observed only at TP1 (Figures S3F and S3G). Together, our results with HGS2 tumor-bearing mice show that the TME of this model responds weakly and transiently to chemotherapy.

### Integration of RNA-seq profiles with ECM structure metrics reveals strategy to target HGSOC tumors with reduced chemo-sensitivity

**Figure 4:**
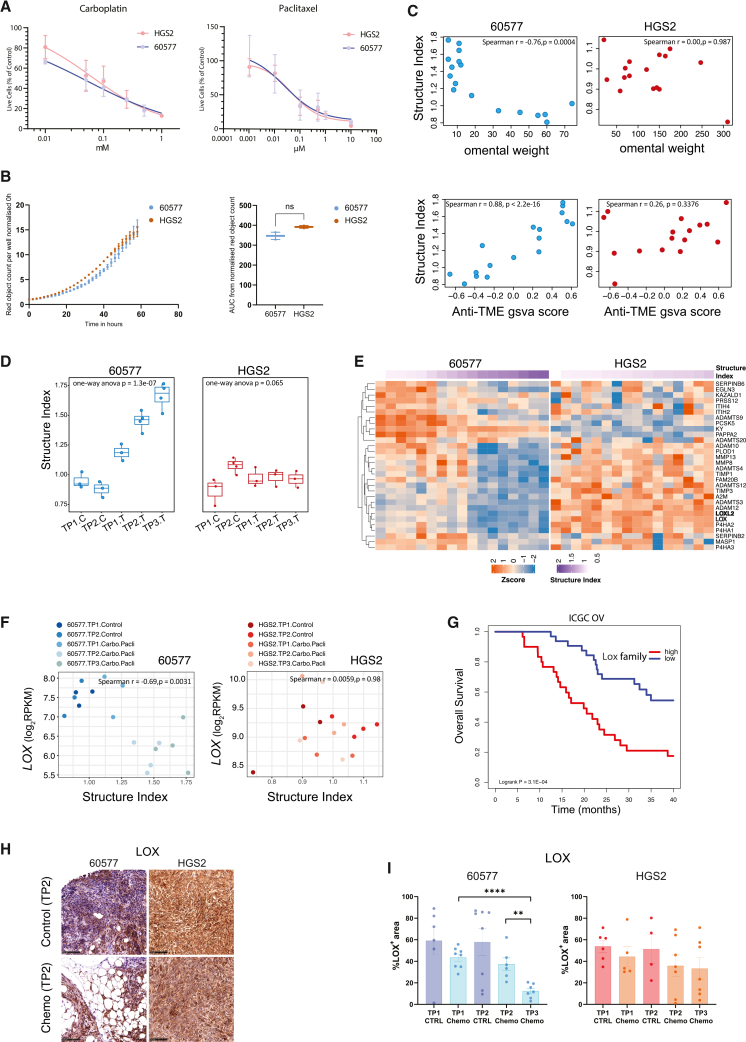
Integration of RNA-seq profiles with IHC metrics uncovers therapeutic targets in chemo-resistant HGSOC (A) Chemo-sensitivity of 60577 and HGS2 mouse cell lines to carboplatin and paclitaxel. The ratio of live cells treated with carboplatin and paclitaxel/live cells from control is shown. The results were plotted as the mean ± SEM of three separate experiments for each experimental condition. IC_50_ was tested with Students’ *t* test and was not significant. (B) Incucyte S3 curves showing the proliferation of HGS2 (red) and 60577 (green) cell lines (left), with area under the curve (AUC) of the curves shown on the right. Red lentivirus-labeled cancer cells were plated (*n* = 2 per cell line), and the plate was imaged every 2 h in the incucyte S3. Error bars represent the mean and SD. AUC of the proliferation curves is on the left (*n* = 2 replicates). Error bars represent the mean and SD. Two-sided unpaired Student’s *t* test. (C) Scatterplots illustrating the correlation of structure index with omental tumor weight (top row) and anti-TME GSVA score (bottom row) in 60577 (blue) and HGS2 (red). Each dot represents an individual mouse. The structure index was calculated using the average of scaled IHC metrics, as described in STAR Methods, and showed a positive correlation with anti-TME GSVA scores and a negative correlation with omental tumor weight. (D) Boxplots illustrating the structure index values in the 5 experimental groups for 60577 (blue) and HGS2 (red); one-way ANOVA *p* values are indicated on the graphs. Each dot represents an individual mouse. (E) Heatmaps of gene expression of matrisome regulators, showing a positive association with structure index in 60577 (Spearman’s r ≥ 0.5 and *p* ≤ 0.05) but not in HGS2 (*p* > 0.05). (F) Scatterplots illustrating the correlation of LOX with structure index in each mouse model, with each dot representing an individual mouse. (G) LOX family gene expression GSVA enrichment scores were calculated in the ICGC-OV dataset. Kaplan-Meier plot of overall survival of Lox family (high) and (low) samples on ICGC-OV; high and low groups correspond to the top 33% and lower 33% samples, respectively, ranked by the decreasing LOX family expression score; *n*_high_ = 31; *n*_low_ = 32. (H) Immunohistochemical staining results. (I) Quantification for LOX in control and chemotherapy-treated tumors from 60577 (indicated with blue) and HGS2 models (indicated with orange). % positive area in the tumor plus stromal area (excluding fat) was quantified in both cases. Data are represented as the mean ± SEM. Each dot represents a mouse, *p* values correspond to Kruskal-Wallis and Mann-Whitney tests for individual time points. ∗∗, *p* < 0.01; ∗∗∗, *p* < 0.001. A representative image from TP2 is shown from each tumor, scale bars: 100 μm.

Although 60577 and HGS2-tumor bearing mice displayed different sensitivity to chemotherapy *in vivo*, 60577 and HGS2 cells were equally sensitive to carboplatin or paclitaxel *in vitro* (Figure 4A) and displayed similar growth rates as monocultures (Figure 4B). This suggested that any difference in chemo response was not due to sensitivity of the malignant cells but was affected by their engagement with, and modification of, the microenvironment. This conclusion was further supported by the finding indicating no significant difference in proliferation levels in the tumors of the two different *in vivo* models, measured by IHC to PCNA, although chemotherapy reduced PCNA staining in both (Figure S4A).

We also found differences in RNA expression levels for integrin receptor and ECM organization genes between the two cell lines grown as monocultures (Figure S4B and Table S4).

As the 60577 and HGS2 cells appear to be equally responsive to chemo, the effects of chemo *in vivo* could be attributed mainly to their interactions with the host. This is supported by differences in TME transcriptomes, ECM structure, fibroblast, and immune cell densities reported in our previous publication^13^ and described above, as well as differences in integrin pathway gene expression in the cell lines.

To identify ECM targets of potential importance to chemo response, we calculated a score, combining the ECM metrics, that correlated with the anti-TME score, and tumor weight as shown in Figures 2C and 2D. We termed this the “structure index.” The structure index reflects features of the ECM that are associated with response to chemotherapy and can be computed for each sample. In 60577, a high structure index denoted an ECM structure linked to a good response to chemotherapy, as shown by its positive correlation with the anti-TME score and negative correlation with omental tumor weight (Figure 4C), while a low structure index associated with a poor response. This correlation was not observed in HGS2 tumors. The structure index increased with time after treatment in 60577 tumors but did not change significantly in HGS2 (Figure 4D).

This gave us a score that we could integrate with transcriptomic data in each tumor. We focused on matrisome genes. Genes correlated with the structure index, and, therefore, also correlated with the ECM structure and response to chemo. 198 and 160 matrisome genes, respectively, were associated with a significant increase and decrease in structure index with chemo in 60577, and these included all six different categories of ECM genes (Figure S4C). We constructed heatmaps of those genes, selecting those that significantly decreased with chemotherapy in 60577 (as the structure index increased) but were largely unchanged in HGS2 (Table S5).

Because the expression of individual genes in these heatmaps correlated with the ECM structure and response to chemo, we reasoned this analysis might identify genes whose protein products could be targeted to generate ECM changes and improve response to chemotherapy. We focused on the ECM regulatory genes as potential targets for proof-of-concept (Figure 4E), selecting lysyl oxidase (LOX) and LOXL2. LOXs are a family of secreted copper-dependent enzymes with important roles in crosslinking of fibrillar collagens into mature ECM.^22^^,^^23^^,^^24^ Untreated tumor gene expression levels of LOX and LOXL2 were lower in 60577 than in HGS2 and showed a significant negative correlation with the structure index in treated 60577 tumors but not in HGS2 (Figures 4F and S4D). In addition, high expression of LOX family genes correlated with lower overall survival in the ICGC ovarian cancer database^25^ (Figure 4G). There was a significant decline in LOX and LOXL2 in response to chemo in 60577 tumors but not in HGS2 (Figures 4H, 4I, S4E, and S4F). LOX was strongly linked to the regulation of ECM genes in a matrisome-based correlation network (Figure S4G). Moreover, in 60577 tumors, chemotherapy induced COL1A1 structure changes that were sustained at TP3 (Figure 2B). As we did not observe such alterations in HGS2, we hypothesized that targeting collagen crosslinking by inhibiting LOX in HGS2 tumors would enhance the response to chemotherapy.

### Early exposure to PXS-5505 increases survival in HGS2 tumor-bearing mice when combined with chemotherapy

**Figure 5:**
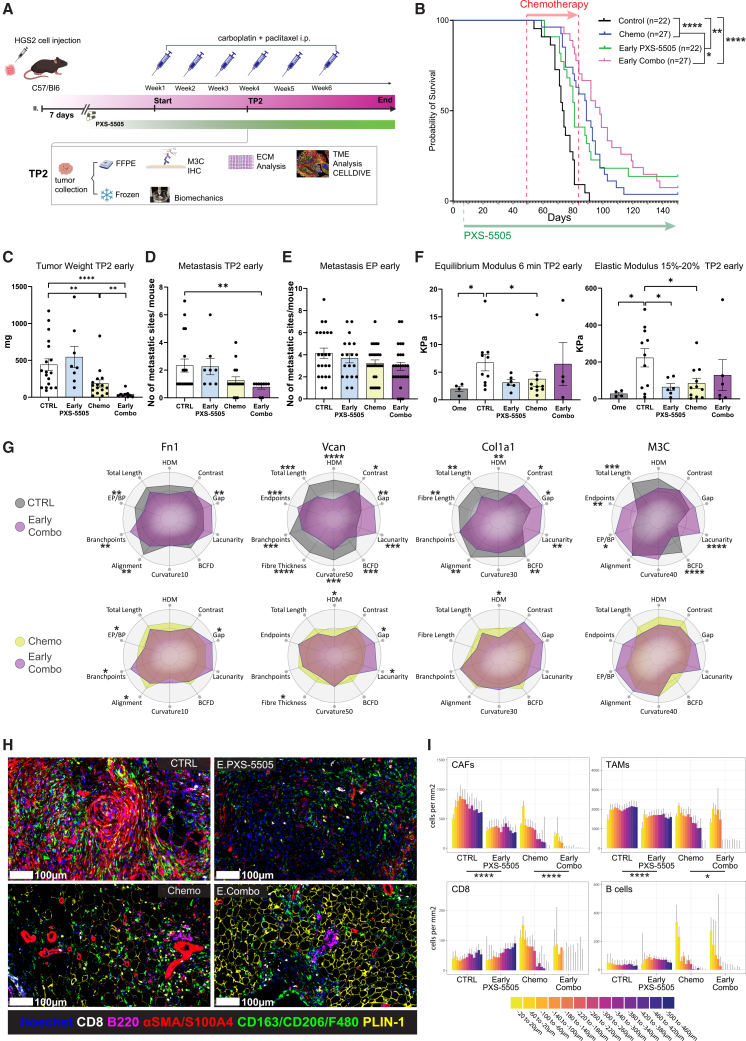
Prolonged exposure to PXS-5505, in combination with chemotherapy, increased survival in HGS2 tumor-bearing mice (A) Schematic of the *in vivo* experiment for HGS2 survival and the experimental outcomes at TP2. C57/Bl6 mice were injected i.p. with HGS2 cells and treated with 20 mg/kg carboplatin and 10 mg/kg paclitaxel or vehicle once per week for three (TP2) or six cycles (endpoint). Onset of chemotherapy treatment commenced 49 days post tumor cell injection, whereas onset of PXS-5505 treatment commenced *ad libitum* 7 days post tumor cell injection (early treatment), where we have found colonization of omentum by HGS2 tumor cells and continued to endpoint. Estimated dose of PXS-5505 *ad libitum* was 170 mg/kg LOXi, which is equivalent to a 10 mg/kg oral gavage. (B) Response of mice injected with HGS2 to six cycles of chemotherapy and/or to early treatment with PXS-5505 *ad libitum*. Median survival times for the control, chemo, early PXS-5505, and early Combo are 73, 89, 81, and 98 days, respectively. The log-rank *p* value is depicted on the survival curves; ∗, *p* < 0.05; ∗∗, *p* < 0.01; ∗∗∗∗, *p* < 0.0001. The start of the treatment is indicated by the red arrow for chemotherapy and the green arrow for PXS-5505. Number of mice in each arm is in parentheses; data were pooled from three independent experiments. (C–E) Quantification of total tumor weight, metastasis analysis at TP2 and endpoint (EP) across all treatment groups, following treatment plan depicted in (A). Data are derived from one experiment combined with data from control and chemotherapy-treated mice from two additional independent experiments. Data are shown as the mean ± SEM, and *p* values correspond to two-tailed Mann-Whitney U test; ∗, *p* < 0.05; ∗∗, *p* < 0.01; ∗∗∗∗, *p* < 0.0001. Overt metastatic lesions in different anatomical regions were recorded, with each dot representing one mouse; control, *n* = 18; chemo, *n* = 16, early PXS-5505, *n* = 8; and early combo, *n* = 9. (F) Biomechanical assessment of control and early treated HGS2 tumors at TP2. Equilibrium modulus as well as elastic modulus are within the range of 15%–20% and were calculated for TP2 across all treatment groups following treatment plan as depicted in (A). *Ome (normal omentum), n* = 4; ctrl (control), *n* = 10 (Equilibrium Modulus), n = 11 (Elastic Modulus); early PXS-5505, *n* = 6 (for both); chemo, n = 10 (Equilibrium Modulus), n = 11 (Elastic Modulus); early combo, *n* = 4 (Equilibrium Modulus), n = 6 (Elastic Modulus). *p* values correspond to two-tailed Mann-Whitney U test; ∗, *p* < 0.05. (G) Structural and textural modifications of ECM induced by early combination treatment with PXS-5505 and chemo in HGS2 tumors at TP2 compared with control and chemo. Radar plots show selected metrics for FN1, VCAN, COL1A1, and M3C fibers. The scaled average for each group is depicted. Control, gray; early combo, purple; chemo, yellow. *p* values correspond to two-tailed Mann-Whitney U test, ∗, *p* < 0.05; ∗∗, *p* < 0.01; ∗∗∗*p* < 0.001; ∗∗∗∗*p* < 0.0001. Data pooled from two individual experiments, along with control and chemo-treated samples from a third experiment, are shown; control, *n* = 16–18; chemo, *n* = 16; early combo, *n* = 9. (H) Representative images of tumors from each treatment group stained for tumor-associated macrophages (TAMs, CD163/CD206/F480, green), adipocytes PLIN1 (yellow), CD8 (white), B220 (fuchsia). and cancer-associated fibroblasts (CAFs, αSMA/S100A4, red). Hoechst 33342 is in blue; scale bars are 100 μm. (I) The distribution of CAFs, TAMS, CD8, and B cells was recorded using the infiltration analysis tool from HALO in 40-μm wide zones from malignant cell areas in all four treatment groups, over a 500-μm range. Data are shown as the mean + SEM, *p* values correspond to two-way ANOVA of cell density comparing treatment groups, taking zones into account. ∗, *p* < 0.05; ∗∗∗∗, *p* < 0.0001.

We tested our hypothesis using a recently described ECM modulating pan-LOX inhibitor PXS-5505^26^ that inhibits *de novo* collagen crosslinking. We, first, treated tumors at an early stage of development to assess if preventing the build-up of excessive collagen crosslinking would enhance the effects of chemotherapy. We started oral treatment with the LOX inhibitor in chow seven days after HGS2 cell injection, commencing chemotherapy six weeks later (Figure 5A). Mice were administered six doses of chemo to replicate the treatment regime of most HGSOC patients.^7^ Combining results from three experiments, we found that six cycles of chemotherapy resulted in an increase in mouse median survival by two weeks (73 versus 89 days) (Figure 5B). “Early” treatment with PXS-5505 alone had a small but significant effect on mouse survival (73 versus 81 days, *p* = 0.002), with three long-term survivors (two at 203 days, one at 405 days) (Figure 5B). PXS-5505 treatment also significantly reduced the levels of proliferation in the tumors, as measured by PCNA staining, at TP2 (Figure S5A). Early PXS-5505 treatment also significantly enhanced the effects of chemotherapy, although the improvement in survival was modest (89 versus 98 days, *p* = 0.05; Figure 5B).

We analyzed tumors one week after the third dose of chemo, equivalent to TP2, with these treatment combinations. We chose TP2 as this time point displayed the strongest ECM structure changes in 60577 chemo-treated tumors. Early PXS-5505 treatment enhanced the tumor weight reduction caused by chemo (Figure 5C), and the number of metastases was significantly reduced in the combination group compared with control (Figure 5D), but there was no difference in the numbers of metastases at endpoint (Figure 5E).

We evaluated tumor stiffness by using stress relaxation tests with unidirectional indentation, calculating both the elastic modulus at 15%–20% strain and the equilibrium modulus. Elastic modulus reflects tissue stiffness under moderate, physiologically relevant deformation, providing insights into the ECM structure, disease state, and treatment response.^27^ Higher values often indicate fibrosis or matrix crosslinking, poor drug penetration, or immune exclusion,^28^ while lower values suggest ECM softening or remodeling.^29^ Higher values of equilibrium modulus indicate persistent matrix stiffness, while lower values suggest degradation or therapeutic remodeling.^27^^,^^28^ Elastic modulus was significantly reduced with early PXS-5505 treatment and with chemo (Figure 5F). We next stained the tumors for FN1, VCAN, COL1A1, and fibrillar collagens. There were no changes in protein density, apart from VCAN that decreased with chemo—a change that was sustained in the combination treatment (Figure S5B). Early PXS-5505 treatment alone altered M3C and FN1 structures relative to control, further altered VCAN, and reduced COL1A1 HDM in the combination treatment compared with chemotherapy (Figures 5G and S5B). It was interesting to note some changes to the ECM in the chemo-treated HGS2 tumors had similarities to the 60577 responses described earlier (Figures 2B and S5C).

As chemo had also altered the transcriptional patterns of immune cells in 60577, we used spatial multiplex IHC to study the HGS2 TME with a total of seventeen different markers. Representative images with a selection of markers are shown in Figures 5H and S5D. Measuring the density of TME cells in the 500 μm region from the outer edge toward the malignant cell area, we noted a significant reduction in the density of CAFs (aSMA^+^ and/or S100A4^+/−^ and CK14 and 19 and F4/80) and tumor-associated macrophages (TAMs, CD206+ and/or CD163^+^ and F4/80^+^ and aSMA^−^) with early PXS-5505, both as monotherapy and in combination with chemotherapy (Figure 5I), while B and CD8^+^ T cell numbers and distribution remained unchanged.

In conclusion, if given early during tumor development, PXS-5505 alone altered the TME of established tumors, reducing stiffness and opening up the space between ECM fibers. It also reduced the distribution and density of fibroblasts and TAMs and enhanced survival when combined with chemotherapy.

### LOX inhibition in established tumors transiently decreased stiffness and altered ECM structure and the immune infiltrate but did not deliver a sustained response when combined with chemotherapy

**Figure 6:**
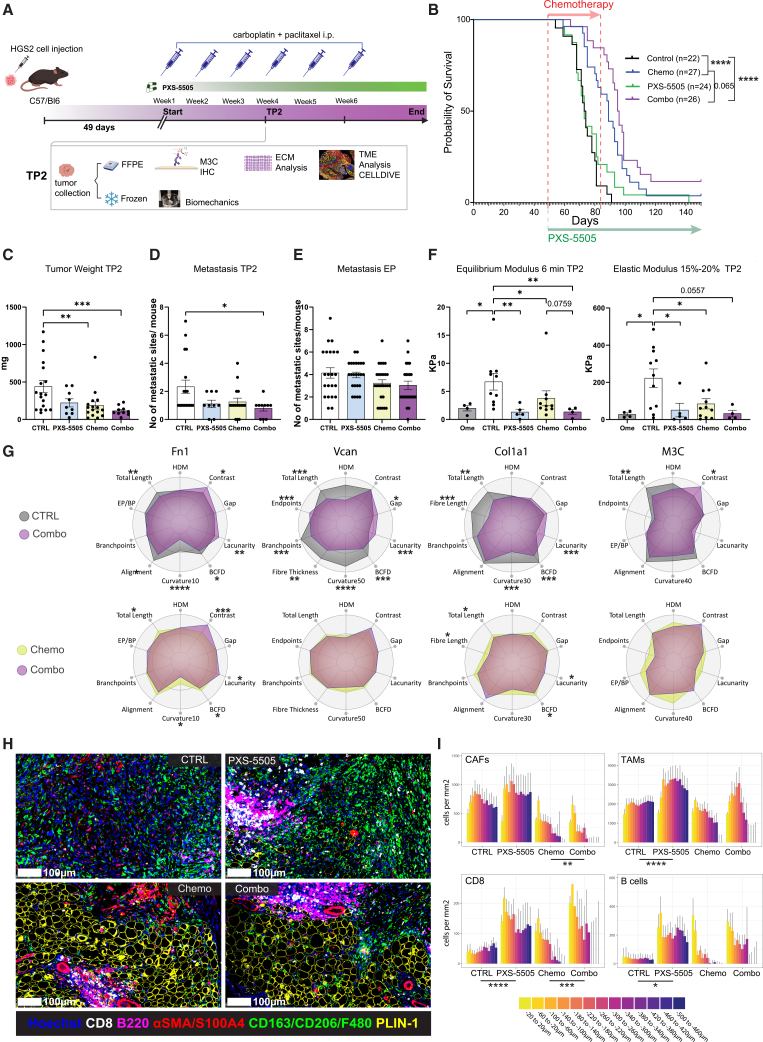
Concomitant chemotherapy and LOX inhibition decreased tumor stiffness and altered ECM structure and immune infiltration (A) Schematic of *in vivo* HGS2 survival and time point (TP2) study. C57/Bl6 mice were injected i.p. with HGS2 cells and treated with 20 mg/kg carboplatin and 10 mg/kg paclitaxel once per week or vehicle for three (TP2) or six cycles (endpoint) and/or PXS-5505. Onset of chemotherapy (i.p.) and PXS-5505 (*ad libitum*) treatment commenced with first detection of palpable tumors (49 days post tumor cell injection). Estimated dose of PXS-5505 *ad libitum* was 170 mg/kg LOXi, which is equivalent to a 10 mg/kg oral gavage. M3C = Masson’s Trichrome. (B) Response of mice injected with HGS2 cells to six cycles of chemotherapy and/or treatment with PXS-5505 *ad libitum*. Survival curve is shown and median survival for control is 73days, chemo 89days, late PXS-5505 73days, combo 95 days. The log-rank *p* value is depicted on the survival curves; ∗∗∗∗, *p* < 0.0001. The start of the treatment is indicated by the red arrow for chemotherapy and the green arrow for PXS-5505. Number of mice enrolled in each arm is shown in parentheses. Data pooled from three independent experiments are shown. (C–E) Quantification of total tumor weight at TP2, metastasis analysis for TP2 and endpoint (EP), respectively. Overt metastatic lesions in different anatomical regions were recorded, with each dot representing one mouse; control, *n* = 18; chemo *n* = 16; late PXS-5505, *n* = 9; combo, *n* = 10. Data were derived from two independent experiments combined with data from control and chemotherapy- treated mice from a third experiment. Data are shown as the mean ± SEM. *p* values correspond to a two-tailed Mann-Whitney U test; ∗, *p* < 0.05; ∗∗, *p* < 0.01; ∗∗∗, *p* < 0.001. (F) Biomechanical assessment of the control and treated HGS2 tumors. Equilibrium modulus and elastic modulus are within the range of 15%–20% and were calculated for TP2 across all treatment groups following treatment plan as depicted in (A). Each dot represents a tumor or tissue from an individual mouse. *p* values correspond to a two-tailed Mann-Whitney U test; ∗, *p* < 0.05; ∗∗, *p* < 0.01. *Ome (normal omentum), n* = 4; ctrl (control), *n* = 10 (Equilibrium Modulus), n = 11 (Elastic Modulus); PXS-5505, *n* = 5 (for both) ; chemo, n = 10 (Equilibrium Modulus), n = 11 (Elastic Modulus); combo, *n* = 4 (for both). (G) Structural and textural modifications of ECM induced by late combination treatment with PXS-5505 and chemo in HGS2 tumors at TP2 compared with control and chemo. Radar plots computed as previously, showing selected metrics for FN1, VCAN, COL1A1, and M3C fibers. The scaled average for each group is depicted. Control, gray; late combo, purple; chemo, yellow. *p* values correspond to a two-tailed Mann-Whitney U test; ∗, *p* < 0.05; ∗∗, *p* < 0.01; ∗∗∗, *p* < 0.001; ∗∗∗∗, *p* < 0.0001. Data pooled from two individual experiments are shown, along with controls and chemo-treated samples from a third experiment; control, *n* = 16–18; chemo, *n* = 16; late PXS-5505, *n* = 8; late combo, *n* = 10. (H) Representative image panel of a tumor from each treatment group stained for tumor-associated macrophages (TAMs, CD163/CD206/F480, green), PLIN1 (yellow), CD8 (white), B220 (fuchsia) and cancer-associated fibroblasts (CAFs, αSMA/S100A4, red). Hoechst 33342 is in blue; scale bars are 100 μm. (I) The distribution of CAFs, TAMS, CD8, and B cells was recorded using the infiltration analysis tool from HALO in 40-μm wide zones from malignant cell areas in all four treatment groups over a 500-μm range. Data are shown as the mean ± SEM, *p* values correspond to two-way ANOVA of cell density comparing treatment groups and taking zones into account; ∗, *p* < 0.05; ∗∗, *p* < 0.01; ∗∗∗, *p* < 0.001; ∗∗∗∗, *p* < 0.0001.

To mimic the combination of PXS-5505 with chemotherapy in a more clinically relevant setting, we assessed whether PXS-5505 treatment of established tumors, commencing concomitantly with chemotherapy seven weeks after HGS2 cell injection, also enhances the effect of chemotherapy (Figure 6A). We combined the results from three independent experiments, two of which had both adjuvant (early) and concomitant (late) PXS-5505 arms; therefore, the control and chemotherapy arms are the same as in Figure 5B. We measured a decrease in LOX activity in the aortas of PXS-5505-treated mice, confirming that drug delivery in chow was effective (Figure S6A). Individual experiments from Figures 5 and 6 are shown in Figure S6B. While PXS-5505 monotherapy did not affect median survival (Figure 6B) or proliferation, as measured by PCNA staining (Figure S6C), there was a non-significant improvement in survival when it was given with chemotherapy relative to chemotherapy alone (95 days versus 89 days; *p* = 0.065, Figure 6B).

PXS-5505 alone did not affect tumor weight, and chemo reduced tumor weight, but the addition of PXS-5505 did not significantly improve this at TP2 (Figure 6C). While chemotherapy alone did not change the number of peritoneal metastatic sites at TP2, there was a significant reduction comparing control to combination therapy (Figure 6D). At endpoint, there was no difference in metastases between the groups (Figure 6E). As with the early treatment, PXS-5505 significantly reduced tumor stiffness, as measured by equilibrium and elastic modulus. This was sustained in the combination group (Figure 6F). We stained the TP2 tumors for FN1, VCAN, Col1A1, and fibrillar collagens. There were no changes in protein density, apart from VCAN that decreased with chemo—a change that was sustained with the combination treatment (Figure S6D). Structural and textural analyses revealed that PXS-5505 treatment led to significant changes in Col1A1, with shorter fibers organized in a less complex pattern (lower BCFD), and an increase in lacunarity, suggestive of increased number and size of gaps in the matrix. Additionally, COL1A1 and FN1 fibers displayed a reduction in curvature, and there was a reduction in HDM in M3C-stained tumors (Figures 6G and S6E). The COL1A1 changes were also seen in the PXS5505 group as well as well as some of the FN1 features (Figure S6E). Collectively, these alterations of the ECM structure are consistent with the tumor stiffness reduction observed in Figure 6F.

Using multiplex IHC and spatial analysis to study the TME (Figures 6H and S6F), we measured the distribution of cells in a 500 μm area from the edge to the tumor center. There was a significant increase in CD8^+^ T cells and B220^+^ B cells with PXS-5505 treatment at TP2 (Figure 6I). The CD8 increase was also significant in the combination treatment arm compared with that in chemotherapy alone. TAMs were significantly increased in PXS-5505-treated tumors compared with control. In addition, CAFs displayed a significant change in their distribution in the combination treatment arm compared with chemotherapy alone, with lower densities observed in the combination treatment arm (Figure 6I).

Therefore, although PXS-5505 alone did not significantly influence mouse survival or metastatic spread, this oral treatment successfully changed the structure and density of collagen, along with other ECM molecules, and reduced tumor stiffness—alterations that were largely retained when PXS-5505 was combined with chemotherapy. In addition, PXS-5505 alone and in combination with chemotherapy increased CD8, B cell, and TAM infiltration in the established tumors.

## Discussion

The effects of therapy on the TME have often been studied at a single time point, usually after NACT. The results from our study show, for the first time, the dynamic transcriptional and ECM protein changes over a period of twenty-one days in mouse HGSOC models after three doses of chemotherapy. In 60577 tumors, a chemo-sensitive model, chemotherapy altered the structure of several key ECM molecules, resulting in a looser and more open ECM. Chemotherapy induced dynamic changes in ECM regulation and innate and adaptive immune response pathways at the RNA level. Furthermore, chemotherapy-induced immune pathway changes in 60577 tumors showed significant overlap with those previously reported in responsive human HGSOC patients,^15^ showing relevance of our mouse experiments to human cancers. In HGS2 tumors, a partially responsive model, chemotherapy only induced weak and transient transcriptomic and ECM structure changes. If immune cell signatures were altered during response to chemotherapy, these changes were not maintained.

The anti-TME signature we adapted from the Bagaev et al. paper^14^ correlated with omental tumor weight and was, therefore, a good metric to assess response to chemotherapy in our mouse models. Using this signature, we showed that several ECM structural and textural metrics correlated with response to chemotherapy. Some of these also associated with PFS in human HGSOC, further supporting our conclusion that ECM structure impacts the response to chemotherapy. Combining the metrics associated with chemotherapy response, we computed the structure index, which summarized features of the ECM that correlated with response to chemotherapy. Thus, the structure index was a measurement of chemo-responsive ECM. Matrisome genes whose expression correlated with the structure index were involved in building or maintaining a chemo-responsive ECM, and those that were altered in 60577, but not in HGS2, were potential therapeutic targets.

The data in this paper and our previous publication^13^ suggest that the distinct TMEs that develop as the tumors grow are largely responsible for their response to chemo. The differences in 60577 and HGS2 cell line expression of MMPs and integrin pathways further supports this notion.

This study reports a list of matrisome gene targets that may be involved in fostering ECM features associated with chemo response. Among these, we chose to further investigate LOX and LOXL2 because their mRNA levels associated with a poor prognosis in patients and with response to chemotherapy in our mouse models. LOX and LOXL2 were also involved in an extensive regulatory network of many other ECM-related genes in our transcriptional dataset.

Prolonged exposure of HGS2 tumor-bearing mice to a highly selective pan-LOX inhibitor, PXS-5505, from an early stage of development confirmed our hypothesis that preventing the build-up of excessive collagen crosslinking would enhance the effect of chemotherapy both in the reduction of tumor burden and metastasis in the early stage and thus increase survival, although this effect was modest. The increase in median survival is consistent with the results of previous studies showing that PXS-5505 enhanced the effect of gemcitabine in a mouse model of pancreatic cancer,^26^ as well as the effect of fluorouracil and oxaliplatin (FOX) in intrahepatic cholangiocarcinoma.^30^ Unlike the pancreatic cancer study, in our HGS2 model, three doses of carboplatin and paclitaxel treatment did not induce fibrosis. Since PXS-5505 inhibits *de novo* collagen crosslinking, we suggest that the treatment of established tumors in the absence of chemotherapy-induced fibrosis limits the treatment’s efficacy. In addition, PXS-5505 was given *ad libitum* in chow. We observed that the mice ate less following chemotherapy or in advanced stages of the disease (data not shown), which restricted PXS-5505 intake and accounts for the lack of effect on survival.

However, PXS-5505 had significant effects on the TME that could be relevant to its clinical use. Given alone or in combination with carboplatin/paclitaxel to the established peritoneal tumors, we found significant modifications of ECM structure, concomitant with a decrease in tumor stiffness and an increase in CD8^+^ and B cell infiltration. This was mitigated by an abundance of fibroblasts and CD163^+^ macrophages which may have suppressed any anti-tumor activity. However, when PXS-5505 was given as tumors developed, the distribution and density of TAMs and fibroblasts significantly decreased, which may have contributed to the improvement in survival with PXS-5505 alone and the enhancement of the chemotherapy response. On the other hand, early PXS-5505 treatment did not increase B cell and CD8^+^ T cell infiltration. This could be due to an insufficient decrease in elastic modulus, which has previously been associated with immune cell infiltration,^28^^,^^29^ and we cannot rule out that a transient increase in B cells and CD8^+^ T cells occurred at an earlier time point. The reduction in TAMs and fibroblasts in these tumors and the increase in B cells and T cells in the advanced tumors suggest that this treatment combination has the potential of creating a more favorable TME, which may be more responsive to immunotherapy can be beneficial. In support of this, an extensive study in several mouse models showed how another LOX inhibitor improved T cell migration into tumors and that its combination with anti-PD1 improved survival in a pancreatic cancer model.^31^ Collectively, the structural and TME changes associated with PXS-5505 suggest a greater penetration of chemotherapy in tumors; thus, an adjuvant PXS-5505 treatment regime could be beneficial to patients. Overall, our results demonstrate that targeting the ECM has the potential to enhance the effect of chemotherapy in HGSOC and that further combination with immunotherapy could be beneficial to patients.

### Limitations of the study

We acknowledge that there are certain limitations in this study. For instance, the number of experimental models analyzed was limited. Future studies incorporating a broader panel of models, including matched chemo-sensitive and -resistant models sharing a common background, would enable more controlled comparisons. Furthermore, analysis of a larger cohort of human samples, especially from patients with well-characterized and robust responses to chemotherapy and prolonged survival, may improve assessment of the ECM topography-associated phenotypes and could reveal closer concordance with the observations made in mouse models.

## Resource availability

### Lead contact

Further information and requests for resources and reagents should be directed to and will be fulfilled by the lead contact, Frances Balkwill (f.balkwill@qmul.ac.uk).

### Materials availability

This study did not generate new unique reagents.

### Data and code availability

• The data generated in this study are available as supplemental spreadsheets and in the key resources table and are also available upon request from the corresponding author. The transcriptomic data are publicly available in Gene Expression Omnibus (GEO) under accession numbers GEO: GSE227100 and GSE270878.
• This paper does not report original code. We have not generated any new software, algorithm, or unpublished custom code. We have provided references for all software used in this study in the key resources table and STAR Methods.
• Any additional information required to reanalyze the data reported in this work paper is available from the lead contact upon request.
• All other data reported in this manuscript will be available by the lead contact upon request.

## Acknowledgments

This project was funded by 10.13039/501100000289Cancer Research UK program grants A16354 and A25714 to F.R.B., C.B., P.K., F.L., and E.M.; UKRI Frontier Research grant (no. EP/X028704/1) to F.R.B., B.M., J.D.J., R.C.B.-R., and E.M.; Wellbeing of Women ELS960, RTF1013, Barts Charity seed fund G-002595, and City of London Development Fund CTRQQR-2021\100004 to S.E. J.H. and E.M. received funding from City of London CRUK Core Award CTRQQR-2021\100004. R.M. received funding from the Barts Charity Grant ECMG1B6R. PXS-5505 was conceived and developed by Pharmaxis. Pharmaxis has been solely responsible for the development of the PXS-5505 compound using their own funds. PXS-5505 was provided free of charge. We would like to thank the surgeons from Barts Trust the St. George’s Hospital Trust and the Barts Cancer Institute (BCI) Tissue Bank. We would also like to thank Miss Anna Malliouri for her preliminary work with TWOMBLI, Dr Luke Gammon, and the Blizard Phenotypic Screening Facility for assistance with our work on Cell DIVE; the Biological Services Unit, Mr. Jordan Chattenton, the Animal Technician Service at BCI, and especially Mr. Colin Pegrum, Mr. James Cormack, Mr. Hagen Schmidt, and Miss Harmony Blythin for assistance and support with *in vivo* experiments. We would also like to thank Nadia Rahman and the BCI Pathology Core, Drs. Linda Hammond, and Sam Wallis from the BCI microscopy core facility (CRUK microscopy core service grant at Barts Cancer Institute, CRUK Core Award CTRQQR-2021\100004). Most importantly, we express our gratitude to the patients for donating the samples, without which this work would not have been possible.

## Author contributions

P.K., F.L., E.M., and F.R.B. designed the project; P.K. and F.L. designed, performed, and analyzed the mouse experiments and mouse and human tissues; E.M. designed and performed the bioinformatics analyses; E.D.F. and P.K. performed indentation analysis; P.K., F.L., and J.H. performed multiplex immunofluorescence on mouse tissues; L.P. and W.J. performed the aorta assay and provided the chow for the mouse experiments with PXS-5505; C.B., S.E., J.D.J., B.M., and R.C.B.-R. performed and analyzed *in vitro* experiments; S.E., C.B., L.P., W.J., and J.F.M. made intellectual contributions; R.M. provided patient materials and intellectual input; F.L., P.K., E.M., and F.R.B. wrote the manuscript. All authors edited and commented on the manuscript.

## Declaration of interests

W.J. and L.P. are employees and shareholders of Syntara (previously, known as Pharmaxis). Syntara (Pharmaxis) provided PXS-5505 free of charge for the work presented here. F.R.B. has received honoraria from Glaxo Smith Klein. R.M. declares honorarium for advisory board membership from Astrazeneca, Merck Sharp and Dohme, and Everything Genetics Ltd.

## STAR★Methods

### Key resources table

**Table:** 

REAGENT or RESOURCE	SOURCE	IDENTIFIER
**Antibodies**		

Alexa Fluor® 488 Anti-CD4 antibody [EPR19514]	Abcam	Cat# ab277275; RRID: AB_2686917
Alexa Fluor® 488 Anti-S100A4 antibody [EPR14639(2)]	Abcam	Cat# ab208566; RRID: AB_2728774
Alexa Fluor® 555 Anti-Cytokeratin 19 antibody [EP1580Y]	Abcam	Cat# ab203444; RRID: AB_2857974
Alexa Fluor® 647 Anti-CD163 antibody [EPR19518]	Abcam	Cat# ab313666; RRID: AB_2943126
Alexa Fluor® 647 Anti-Fibronectin antibody [EPR23110-46]	Abcam	Cat# ab313350; RRID: AB_2941028
alpha-Smooth Muscle Actin Antibody (1A4/asm-1) [Alexa Fluor® 750]	Novus	Cat# NBP2-33006AF750; RRID: AB_3282905
anti-Fibronectin antibody produced in rabbit	Sigma-Aldrich	Cat# F3648; RRID:AB_476976
Anti-Goat IgG (H + L), made in horse	Vector Laboratories	Cat# BA-9500; RRID: AB_2336123
anti-LOX antibody (EPR4025)	Abcam	Cat# ab174316; RRID: AB_2630343
Anti-Mouse IgG (H + L), made in goat	Vector Laboratories	Cat# BA-9200; RRID: AB_2336171
Anti-PCNA antibody [PC10] (Alexa Fluor® 647)	Abcam	Cat# ab201674; RRID: AB_2857977
Anti-Rat IgG (H + L), mouse adsorbed, made in goat	Vector Laboratories	Cat# BA-9401; RRID: AB_2336208
anti-VCAN antibody produced in rabbit	Sigma-Aldrich	Cat# HPA004726; RRID:AB_1080561
CD45R/B220	BD Biosciences	Cat# 553089; RRID: AB_394619
COL1A1 (E8F4L) Rabbit Monoclonal Antibody (Alexa Fluor® 647 Conjugate) #72827	Cell Signaling Technology	Cat# 72827; RRID: AB_3696808
Collagen I antibody	Abcam	Cat#ab21286; RRID: AB_446161
Donkey Anti-Goat IgG H&L (Alexa Fluor® 750) preadsorbed	Abcam	Cat# ab175745; RRID: AB_2924800
Donkey anti-Rabbit IgG (H + L) Highly Cross-Adsorbed Secondary Antibody, Alexa Fluor™ Plus 647	Thermo Fisher Scientific	Cat# A32795; RRID: AB_2762835
Endomucin (V.7C7) PE conjugated	Santa Cruz Biotechnology	Cat# sc-65495; RRID: AB_2100037
F4/80 (D2S9R) XP® Rabbit mAb	Cell Signaling Technology	Cat# 25514; RRID: AB_2799771
FOXP3 Monoclonal Antibody (FJK-16s), PE, eBioscience	Thermo Fisher Scientific	Cat# 12-5773-82; RRID: AB_465936
Goat Anti-Mouse Mmr Polyclonal antibody, Unconjugated	R and D Systems	Cat# AF2535; RRID: AB_2063012
Goat Anti-Rabbit IgG Antibody (H + L), Biotinylated	Vector Laboratories	Cat# BA-1000; RRID: AB_2313606
LOXL2 antibody	Abcam	Cat# ab96233; RRID: AB_10677617
Mouse IgG1 kappa Isotype Control (P3.6.2.8.1), eBioscience	Thermo Fisher Scientific	Cat# 14-4714-82; RRID: AB_470111
Mouse IgG2a kappa Isotype Control (eBM2a), eBioscience	Thermo Fisher Scientific	Cat# 14-4724-82; RRID: AB_470114
PCNA (D3H8P) XP® Rabbit mAb #13110	Cell Signaling Technology	Cat# 13110; RRID: AB_2636979
Perilipin-1 (D1D8) Rabbit Monoclonal Antibody (Alexa Fluor® 647 Conjugate) #52975	Cell Signaling Technology	Cat# 52975S; RRID: AB_10829911
Rabbit Anti-Collagen I Polyclonal antibody Unconjugated	Abcam	Cat# ab34710; RRID: AB_731684
Rabbit IgG Isotype Control	Thermo Fisher Scientific	Cat# 31235; RRID: AB_243593
Rat IgG2a kappa Isotype Control (eBR2a), eBioscience	Thermo Fisher Scientific	Cat# 14-4321-81; RRID: AB_470104
Recombinant Alexa Fluor 555 Anti-CD8a antibody (EPR21769)	Abcam	Cat# ab280863; RRID: AB_2927548
Recombinant Anti-Cytokeratin 14 antibody (EPR17350)-Cytoskeleton Marker	Abcam	Cat# ab181595; RRID: AB_2811031

**Bacterial and virus strains**		

IncuCyte NucLight Red Lentivirus Reagent (puro-selectable)	Essen BioScience (Sartorius)	Cat# 4625, Lot number: LSP032219.02–051019

**Biological samples**		

Human HGSOC omental metastasis samples	Barts Health NHS Trust, St. George’s University Hospitals NHS Foundation Trust and Barts Gynae Tissue Bank	(https://directory.biobankinguk.org/Profile/Biobank/GBR-1-128) HTA license number 12199 (REC no: 10/H0304/14 and 15/EE/0151)

**Chemicals, peptides, and recombinant proteins**		

0.5% Trypsin-EDTA (10X)	GIBCO	Cat# 15400-054
10X Casein (250 mL)	Vector Laboratories	Cat# SP-5020
10x TBS Buffer pH 7.4	Severn Biotech Ltd	Cat# 20-7301-10
Animal-Free Blocker and Diluent, R.T.U.	Vector Laboratories	Cat# SP-5035
Antibiotic-Antimycotic (100X)	GIBCO	Cat# 5240-062
Antibody diluent	Vector Laboratories	Cat# SP-5035
Antigen Retrieval Buffer (100X Tris-EDTA Buffer, pH 9.0)	Abcam	Cat# ab93684
Antigen Unmasking Solution, Citric Acid Based	Vector Laboratories	Cat# H-3300-250
Antigen Unmasking Solution, Tris Based	Vector Laboratories	Cat# H-3301-250
Bouin’s Solution	Sigma-Aldrich	Cat# HT10132
Bovine Serum Albumin	Sigma-Aldrich	Cat# A4503
Carboplatin	Hospira	Cat# 61703-0339
Carboplatin	MedChemExpress	Cat# HY-17393
Collagenase from Clostridium histolyticum	Sigma-Aldrich	Cat# C9263
Control mouse diet	Research Diets	Cat# D20011301
Diaminobenzidine substrate-chromogen (Dako Liquid DAB+ Substrate Chromogen System)	Dako	Cat# K3468
Distilled Water	Gibco	Cat# 15230204
DMEM:F12 1:1 with GlutaMax	Gibco	Cat# 31331-028
DMSO	Sigma-Aldrich	Cat#41639
DPX Mountant for histology	Sigma-Aldrich	Cat# 6522
EDTA 0.5M	Thermo fisher	Cat# AM9262
Ethanol Absolute 99.8+%	Fisher Scientific Ltd	Cat# E/0650/DF/17
Ethanol denatured (BioUltra)	Sigma-Aldrich	Cat# 51976-500 ML-F
Ethanol, 99.8%, as ethanol, anhydrous, (denat.with 2% IPA +2% MEK)	Fisher Scientific	Cat# 12367103
Fetal Bovine Serum	Hyclone	Cat# 11521831
Fetal Bovine Serum Heat Inactivated	Gibco	Cat# 10438-026
Formalin solution neutral buffered 10%	Sigma-Aldrich	Cat# HT501128
Gill’s hematoxylin	Sigma-Aldrich Cat	Cat# GHS1128
Glacial acetic acid	Fisher Scientific	Cat# 10021123
Glycerol	Sigma-Aldrich	Cat# G9012-500 ML
HBSS (10X), no calcium, no magnesium, no phenol red	Gibco	Cat# 14185-045
Hematoxylin Solution, Gill No. 1	Sigma -Aldrich	Cat# GHS132
Hoechst 33342, Trihydrochloride, Trihydrate - FluoroPure™ Grade	Thermofisher	Cat# H21492
Horse Serum (10 mL)	Sigma-Aldrich	Cat# H0146-10 ML
Hydrocortisone, g-irradiated, powder	Sigma	Cat# H0135
Hydrogen peroxide solution 30%	Sigma-Aldrich	Cat# H1009-500 mL
Hydrogen peroxide solution 30%	Fisher Scientific Ltd	Cat# H/1800/15
Insulin, Transferrin, Selenium, Sodium Pyruvate Solution (ITS-A)	Gibco	Cat# 51300044
Koliphor EL	Merck Life Science Ltd	Cat# C5135
L-glutamine	Sigma	Cat# G7513-100 ML
Murine Epidermal Growth Factor (EGF)	Sigma	Cat# E4127
Normal Donkey Serum	Merck Life Science Ltd	Cat# S30-100 ML
Normal Goat Serum	Sigma-Aldrich	Cat# G9023-10 ML
Paclitaxel	Hospira	Cat# 61703-0342
Paclitaxel	MedChemExpress	Cat# HY-B0015
Pan-LOXi	Syntara (previously known as Pharmaxis)	PXS-5505
Penicillin-Streptomycin (10,000 U/mL)	Invitrogen	Cat# 15140-122
Phosphate-buffered saline (PBS) (1x)	Gibco	Cat# 141-90-094
Puromycin	InvivoGen	Cat# anti-pr-1
PXS-5505 mouse diet	Research Diets	Cat# D21012504R
RNAlater-ice	Thermo Fisher Scientific	Cat# 4427575
RNase-Free DNase Set (50)	Qiagen	Cat#: 79254
RPMI1640	Gibco	Cat# 21875034
Sodium Hydroxide 1M	Fluidigm	Cat# J/7620/15
Triton X-100	Sigma-Aldrich	Cat# T8787
Tween 20	Sigma-Aldrich	Cat# P1379-250 ML
Tween 20	Sigma-Aldrich	Cat# P7949-500 mL
Weigert’s Iron Hematoxylin Solution	Sigma-Aldrich	Cat# HT1079
Xylene, Certified AR	Fisher Scientific Ltd	Cat# X/0250/17
Xylene, Technical	Fisher Chemical Fisher Scientific	Cat# X/0100/17

**Critical commercial assays**		

anti-rabbit ImmPress HRP	Vector Laboratories	Cat# MP7451; RRID: AB_2631198
anti-rat ImmPRESS-HRP reagent	Vector Laboratories	Cat# MP7444; RRID: AB_2336530
Avidin/Biotin Blocking Kit	Vector Laboratories	Cat# SP-2001; RRID: RRID:AB_2336231
DAB Peroxidase (HRP) Substrate Kit	Vector Labs	Cat# SK-4100
FlexAble CoraLite® Plus 750 Antibody Labeling Kit for Rabbit IgG	ProteinTech/FlexAble	Cat# KFA004; RRID:AB_3095335
High Sensitivity RNA ScreenTape	Agilent	Cat# 5067-5579
MycoAlert PLUS Mycoplasma Detection kit	Lonza	Cat# LT07-710
QIAshredder	Qiagen	Cat# 79654
RNeasy Mini kit	Qiagen	Cat# 74104
Super Sensitive Polymer HRP IHC	BioGenex	Cat# QD430-XAKE
Trichrome Stain (Masson) Kit	Sigma-Aldrich	Cat# HT15-1 KT
VECTASTAIN® ABC HRP Kit	Vector Labs	Cat# PK-4000; RRID: AB_2336818

**Deposited data**		

Chemotherapy-induced extracellular matrix remodeling in HGSOC	This paper	GSE270878
Immunological modifications following chemotherapy are associated with delayed recurrence of ovarian cancer	Adzibolozu et al., 2023^15^	GEO: GSE227100

**Experimental models: Cell lines**		

*Mus musculus:* 60577 cells	Laboratory of Difilippantonio S.	Szabova et al., 2014^32^
*Mus musculus:* HGS2 cells	Laboratory of Frances Balkwill	Maniati et al., 2020^13^

**Experimental models: Organisms/strains**		

C57/Bl6J mice	Charles River	IMSR_JAX:000664
FVB mice	Charles River	IMSR_JAX:001800

**Software and algorithms**		

biorender.com	BioRender	RRID:SCR_018361
biomaRt	Bioconductor	Durinck S et al.,2009^33^; RRID:SCR_019214
ComplexHeatmap	Bioconductor	Gu Z et al., 2016^34^; RRID:SCR_017270
Definiens software	(Definiens AG, Germany)	http://www.astrazeneca.com
DenseNetV2 (HALO AI)	Indica Labs, LLC	https://indicalab.com/halo-ai/ RRID:SCR_018350 (HALO platform)
EdgeR	Bioconductor	Robinson MD et al.,2010^35^; RRID:SCR_012802
fgsea	Bioconductor	Korotkevich G et al., 2019^36^; RRID:SCR_020938
FIJI	Schindelin et al., 2012^37^	https://fiji.sc/; RRID:SCR_002285
ggplot2	CRAN	https://cran.r-project.org/web/packages/ggplot2/index.html; RRID:SCR_014601
gplots	CRAN	https://cran.r-project.org/web/packages/gplots/index.html
GraphPad Prism version 10.1.0 and later ones for Windows, GraphPad Software, Boston, Massachusetts USA	GraphPad Software	https://www.graphpad.com; RRID:SCR_002798
GSVA	Bioconductor	Hänzelmann S et al., 2013^38^; RRID:SCR_021058
HALO AI v3.6.4134	Indica Labs, LLC	https://indicalab.com/halo-ai/ RRID:SCR_018350
HALO Area Quantification FL Algorithm	Indica Labs, LLC	RRID:SCR_018350
HALO Highplex FL Algorithm	Indica Labs, LLC	https://indicalab.com/halo/halo-modules/highplex-fl/; RRID:SCR_018350
HALO v.4.0 and v.4.1	Indica Labs, LLC	RRID: SCR_018350
ImageJ 1.48v and 1.54f image processing and analysis program (NIH, Bethesda, MD) with color threshold and color deconvolution plug-ins	Schneider et al., 2012^39^; Ruifrok and Johnston, 2001^40^	https://imagej.net/ij/ https://imagej.net/plugins/colour-deconvolution; RRID:SCR_003070
Incucyte S3 software rev2022b	Essen BioScience/Sartorius	https://www.sartorius.com/incs; RRID:SCR_023147
limma	Bioconductor	Ritchie ME et al., 2015^41^; RRID:SCR_010943
MATLAB	MathWorks	https://www.mathworks.com/products/matlab.html; RRID:SCR_001622
NPD.view 2.7.25 software	Hamamatsu Photonics	https://www.hamamatsu.com/eu/en/product/type/U12388-01/index.html
Nuclei Seg BF v1.0.0 (HALO AI)	Indica Labs, LLC	https://indicalab.com/halo-ai/ RRID:SCR_018350 (HALO platform)
QuPath versions 0.2.0 to 0.5.0	Bankhead et al., 2017^42^	https://qupath.github.io/; RRID:SCR_018257
R v4.1	–	http://www.R-project.org; RRID:SCR_001905
TWOMBLI plugin	Wershof et al., 2021^20^	https://github.com/wershofe/TWOMBLI; RRID:SCR_027217

**Other**		

4200 TapeStation System	Agilent Technologies	Model: 4200 TapeStation; Cat#: G2991AA
Cell DIVE™ ClickWell Assembly cassettes	Leica Microsystems	Cat# 29626840
Cell DIVE™ Multiplexed Immunofluorescence (MxIF) Platform	Leica Microsystems	Model: CELLDIVE™
GentleMACS Dissociator	Miltenyi Biotec	Cat# 130-093-235
GentleMACS M Tubes	Miltenyi Biotec	Cat# 130-093-236
Hamamatsu NanoZoomer S210 Slide Scanner	–	–
Incucyte® S3 Live-Cell Analysis System	Sartorius (Essen BioScience)	Model: Incucyte S3
Instron 3342 screwdriver mechanical testing frame	Instron, UK	–
SuperFrost Plus™ Adhesion slides	Fisher Scientific	Cat# 5067-5579
Vi-CELL XR Cell Viability Analyzer, BMSB J540	Beckman Coulter	https://www.mybeckman.uk/cell-counters-and-analyzers/vi-cell-xr#:∼:text=Vi%2DCELL%20XR%20Cell%20Analyzer%20Features, Compliance%20and%20Validation

### Experimental model and study participant details

#### Study approval

##### Murine models

All *in vivo* experimental procedures observed the guidelines approved by the ethics committees of QMUL in accordance with Animals (Scientific Procedures) Act 1986 under the Home Office Project licenses PBE3719B3 and PP5394401. For survival experiments, mice were euthanized when they reached humane endpoint as defined in the licenses.

##### Patient samples

Samples were kindly donated by women with HGSOC undergoing surgery at Barts Health NHS Trust and St. George’s University Hospitals NHS Foundation Trust. Tissues deemed by a pathologist to be surplus to diagnostic and therapeutic requirements were collected along with clinical data under the Barts Gynae Tissue Bank HTA license number 12199 (REC no: 10/H0304/14, 15/EE/0151). Patients gave written informed consent, and the study was approved by a UK national review board. Studies were conducted in accordance with the Declaration of Helsinki and the International Ethical Guidelines for Biomedical Research Involving Human Subjects.

#### Animals, *in vivo* models, treatment, and tissue processing

We used two syngeneic mouse models, 60577 and HGS2, which we published before.^13^ HGS2 cancer cells were developed from female C57/Bl6 mice, whereas 60577 from female FvB. C57BL/6 and FvB wild type mice (aged 7 weeks) were purchased from Charles River Laboratories (UK) and acclimatised for 1–2 weeks prior to the initiation of the studies. Only female animals were used throughout this study. Mice were housed under sterile conditions, in individually ventilated cages with a maximum of 5–6 animals per cage, fed with standard chow diet and water *ad libitum*, and maintained on an automatic 12 h light cycle at 22–24°C. All studies were conducted using sterile techniques.

For the orthotopic tumor growth establishment, 60577 and HGS2 mouse cell lines were cultured as before^13^ and trypsinized, washed in medium and resuspended in PBS to 10^7^ cells in 300μl to be injected intraperitoneally (i.p.) in 8-9-week-old FVB mice (60577) or C57BL/6J mice. For chemotherapy treatment, mice were treated with a combination of carboplatin (20 mg/kg, Hospira) + paclitaxel (10 mg/kg, Hospira), both from the pharmacy at St. Bartholomew’s Hospital, London or from Medchemexpress (HY-17393 and HY-B0015, respectively). Carboplatin and paclitaxel were administered to mice via i.p. in 200 μL volume starting respectively 21 days (60577), or 49 days (HGS2) post-cell injection. Vehicle-treated controls received Koliphor® EL (Merck, C5135), 6.1%, EtOH 6.1%, water 14.7%, PBS 73.1%. Mice were treated with chemotherapy or vehicle via i.p. once weekly for three (TP2) to six weeks.

For Pan-LOX inhibition (LOXi) in the HGS2 model, we used PXS-5505 (Pharmaxis, now Syntara) under an MTA agreement. PXS-5505 was provided in the chow (D21012504R, Research Diets) *ad libitum* in a concentration that is approximately 170mg/kg (maximum dose calculated). The control group mice, control chow D20011301 (Research Diets) was provided. After one week of acclimatization, all mice were introduced to Control chow, D20011301. Mice were then introduced to D21012504R PXS-5505 diet after 7 and 49 days for the early and late PXS-5505 treatment, respectively. PXS-5505 treatment continued up to 22 weeks. For combinatorial treatment of PXS-5505 with chemotherapy in HGS2 model, chemotherapy treatment commenced, described as above.

Humane endpoint for mice was defined as a change in general health; specifically, 15% body weight loss over 72 hours or 20% over any time period, any sign of jaundice, hunched posture, or abnormal breathing pattern, signs of distress and/or suffering, as well as signs of ascites or palpable tumors exceeding the license limits. Any tumor interfering with mobility and access to food and water, as well as ulceration or increased secretion of injection site tumors, blood in the stool and or urine defined humane endpoint additionally. Survival assessment of mice was made by the same individual where possible to limit inter-observer variability and occasionally assistance was provided by a trained animal technician who was not directly involved in the experimental design.

For both time point and endpoint analysis, mice were euthanized under CO2 exposure. Once death was confirmed, an incision to expose the gastrointestinal contents was made that was extended further towards the lower limbs bilaterally to create skin flaps which can be pinned down. Omental, splenoportal and lesser omental tumors were harvested, along with mesentery and their weights measured. Omental tumors were cut in half, with one half being snap frozen in dry ice and the other half fixed in 10% neutral buffered Formalin solution overnight. The formalin fixed tumors were paraffin-embedded and sectioned (4 μm), followed by H&E staining, whereas the frozen tumors were subjected to RNAseq or indentation testing. Tumors in other anatomical sites were excised and weighed. Once all tumors were recorded and weighed, internal organs like liver, pancreas, stomach, spleen, and intestines were removed to better visualize the aorta that was subsequently separated from the spine dorsally and the esophagus ventrally. Once isolated, aortas were snap frozen in dry ice and kept at -80C.

### Method details

#### Murine cell line culture and *in vitro* drug response assay

HGS2 and 60577 described previously^13^ were cultured in complete DMEM:F12 1:1 with GlutaMax medium (Gibco) with the addition of 4% heat inactivated FBS (Hyclone, Gibco), 1x Pen/Strep (Invitrogen), 1x insulin/transferrin/selenium (Invitrogen, 51300), 100 ng/ml hydrocortisone (Sigma, H0135), 20 ng/ml murine Epidermal Growth Factor (EGF, Sigma, E4127), 1x antibiotic-antimycotic (Gibco). Cells were trypsinized with 0.05% trypsin-EDTA (Gibco) and regularly tested for Mycoplasma, using the MycoAlert PLUS Mycoplasma Detection Kit (Lonza, LT07-710). For the chemosensitivity assays, the day prior to drug treatment, 60577 and HGS2 cells were plated at a density of 20,000 cells/well, in a 24 well plate, in triplicates. The day after, cells were treated with 1, 0.5, 0.1, 0.01, or 0.001mM of carboplatin (Hospira, 61703-0339), and 1, 0.5, 0.1, 0.01 or 0.001uM paclitaxel (Hospira, 61703-0342). At 48h, cells were harvested with trypsin and counted with a V-cell-counter (VI-CELL XR Cell Viability Analyzer, BMSB J540). The percentage live cells compared to control was calculated.

#### Lentivirus transduction of the murine cell lines to induce stable expression of nuclear red protein, mKate

IncuCyte NucLight Red Lentivirus reagent (Essen Bioscience (Sartorius), 4625) was used to induce a stable expression of the red mKate2 protein in the cancer cells. The recommended multiplicity of infection (MOI) ranges between 3 to 6. The viral titer was 11.47x 10^6^ transduction units (TU) /ml (Lot number: LSP032219.02-051019).

10,000 cells were plated in a 48 well plate and left to grow until they became 30% confluent. The transduction was started by replacing the conditioned medium in the well with 1 mL of transduction medium that contained 3.5μL of lentiviral particles. Cells were cultured for 24 h in an incubator at 37 °C, 95% humidity and 5% CO2. After 24h, the transduction medium was replaced with normal medium and the cells were cultured for 48-72h in an incubator at 37 °C, 95% humidity, and 5% CO2. The lentivirus integrates into the DNA of the cell and produces mKate2 protein which is red fluorescent. Transduced stable cell populations expressing mkate2 protein were generated by puromycin selection puromycin (InvivoGen, ant-pr-1 ). To determine the dose of puromycin, 250,000 cells were plated per well in 12 well plate for 9 wells. One well was used as control well and the cells in the other 8 wells were treated with increasing concentrations of puromycin starting with 0.5μg/ml to 4μg/ml daily for 4 days. The lowest puromycin concentration that killed all cells in the well was chosen for puromycin selection. A dose of 1.5μg/ml was chosen for HGS2 and 4μg/ml for 60577. Lentivirus transduced cell lines were stored in 1ml aliquots of 10% DMSO+90%FBSs in cryovials at -80C and then transferred to liquid nitrogen.

#### Incucyte S3 to study the proliferation of murine cell lines

Red labelled lentivirus transduced 60577 and HGS2 were plated as 20,000 cells per well in 2ml of medium in 12 well plate in duplicates. Cells are left to attach for 3-4h then the plate is moved to be imaged in Incucyte® S3 Live-Cell Analysis System for 72h. 4 images per well were taken every 2h. Red object count per well normalized to the counts at 0h were analyzed using incucyte S3 software v.2018C and later ones. Data was then exported from the software and used in Prism version 10.1.0) to generate AUC for each individual well. The AUC was then used to study statistical significance using unpaired t-test.

#### Lysyl oxidase family activity from the mouse aorta

Lysyl oxidases family activity was measured in the aortas of tumor bearing mice at sacrifice. The aorta underwent an urea based extraction^32^ and then the level of lysyl oxidase activity was measured with a fluorometric activity assay^33^ to confirm lysyl oxidase inhibition. The signal was defined as the enzyme activity in the aorta, while noise is measured in the presence of a high concentration (300 μM) of BAPN to abolish any lysyl oxidase family activity. Signal over noise equal to 1 indicates complete inhibition.

#### Metastasis analysis

Both at timepoint (TP2) and endpoint, mice were assessed for the number of the metastatic sites identified during autopsy. Main metastatic sites recorded included splenoportal fat, lesser omentum, mesentery, peritoneum, perirenal, spinal, liver, diaphragm, ovaries, ovarian fat pad, fallopian tubes, uterus, uterine fat pad, abdominal fat, chest cavity, gastrointestinal tract. Macroscopical pancreas invasion was also counted as a metastatic site. Each metastatic site counts as 1, regardless of the numbers and size of the metastases on that site. The number of total metastatic sites per mouse was calculated and plotted.

#### Histopathology, immunohistochemistry, and morphometry

Omental tumors from both models were dissected, fixed in neutral buffered formalin solution (Sigma, HT501128) for 24 hr, paraffin-embedded and sectioned (4 μm), followed by H&E staining. For immunohistochemistry, FFPE omental sections were heated for 1hr at 60°C and then submerged twice in xylene for 5min. Slides were then gradually re-hydrated by submerging for 2min in each of the following ethanol solutions: 100%, 90%, 70%, 50% and finally in ddH_2_O for 3min. Antigen retrieval was performed as per Table S6A for all staining, apart from FN1. The slides were subsequently washed, treated with 3% H_2_O_2_ (Fisher Scientific, H/1800/15) in PBS for 5min, washed again and blocked with blocking buffer for 1hr. The primary antibody was added in blocking buffer and incubated at ambient temperature (Table S6A). Slides were washed three times, and the primary antibody was detected as described in Table S6A. Color was developed with Diaminobenzidine substrate-chromogen (Dako Liquid DAB+ Substrate Chromogen System), (Dako, K3468) and tissues were counterstained with Gill’s hematoxylin I (Sigma-Aldrich, GHS1128), washed, dehydrated in ethanol and xylene, and mounted in DPX mountant (Sigma-Aldrich, 06522). For COL1A1 staining on mouse tissues, ab21286 from Abcam was used, whereas ab34710 (Abcam) was used for staining COL1A1 on human tissues.

#### Masson’s trichrome staining

To reveal fibrillar collagen deposition on mouse and human omental biopsies, we used the Trichrome Stain (Masson) Kit (HT15-1KT) from Sigma as previously described in.^13^ Briefly, FFPE 4-μm sections were deparaffinized in xylene and gradually re-hydrated by submerging in a graded series of Ethanol solutions in H_2_O. Slides were further hydrated for 3min in ddH_2_O and fixed in Bouin’s solution (Sigma, HT10132) overnight at RT. The next day, sections were washed in running water and ddH_2_O, until the yellow colour disappeared and counterstained in working Weigert’s Iron Hematoxylin Solution (Sigma, HT1079-1SET). Subsequently, sections were washed in running tap water for 2 minutes, rinsed in ddH_2_O and stained in Biebrich Scarlet-Acid Fuchsin for 15 minutes. Sections were then rinsed in ddH_2_O and immersed in working Phosphotungstic/Phosphomolybdic Acid solution for 10-15 minutes, until collagen fibers were not red. Aniline Blue solution was applied next for 30 minutes; sections were rinsed in ddH_2_O and placed in 1% acetic acid for 3 minutes. Finally, sections were dehydrated very quickly in two changes of 90% alcohol, followed by 2 changes of absolute alcohol, cleared in xylene and mounted in DPX (Sigma-Aldrich, 06522).

#### RNA isolation and sequencing

Total RNA was extracted from frozen murine omental tumor samples or cell lines. The samples were transferred into RNAlater-ice (Thermo Fisher Scientific, 4427575) and homogenized using GentleMACS M tubes (Miltenyi, 130-093-236) in RLT buffer on a gentleMACS Dissociator (Miltenyi Biotec, 130-093-235). Samples were further processed using QIAshredders (Qiagen, 79656) and the Rneasy Mini kit (Qiagen, 74104) with on-column Dnase digestion (RNase-Free DNase Set, Qiagen, 79254). RNA quality was analyzed with the 4200 TapeStation System using the High Sensitivity ScreenTape Assay (Agilent, 5067-5579). Library preparation and RNASeq were performed by the Wellcome Trust Centre (Oxford, UK) using NEBNext rRNA Depletion Kit v2 to deplete rRNA species. Sequencing was performed to ∼65 million reads mean depth for tumour tissue samples or ∼50 million reads mean depth for the malignant cell lines on the Illumina NovaSeq6000 plarform, strand-specific, generating 150 bp paired-end reads. RNA-Seq reads were quality trimmed using trimgalore v0.6.5 and were mapped to the mouse genome (mm10, Genome Reference Consortium GRCm38) using STAR v2.7.0f.^34^ Number of reads aligned to the reference genome were counted using rsem v1.3.1 based on the Ensembl annotation GRCm38.^35^ Only genes that achieved at least one read count per million reads (cpm) in at least twenty-five percent of the samples were kept. This led to 16,400 filtered genes in total, 14,031 of which were protein coding genes for mouse tumors or 13,423 and 12,462 for the malignant cell lines respectively. Conditional quantile normalization was performed accounting for gene length and GC content and a log2-transformed RPKM expression matrix was generated.

#### Bioinformatic analysis

Differential expression analysis was performed in Edge R using limma^36^ for the mouse tumor tissues dataset and with DESeq2 for the malignant cell line monocultures. Gene set enrichment analysis was performed for Canonical Pathways (c2.cp.v7.4.symbols.gmt) using R package fgsea and the ranked t-statistic of all sufficiently detected orthologous genes. Enrichment analysis of gene clusters or overlapping genes of interest (over-representation analysis) was performed with R package dnet, using a hypergeometric test. Single sample gene-set enrichment analysis which calculates a gene-set enrichment score per sample was performed using the R package GSVA.^37^ Heatmaps were plotted with R package ComplexHeatmap; scatter plots, barplots and boxplots with R packages gplots and ggplot2. Structure index was obtained by scaling the metrics and calculating the ratio of those parameters that positively correlated with the anti-tumor microenvironment enrichment score over those that negatively correlated with it. The GSE227100 dataset containing RNASeq data of 24 patients with paired pre-NACT and post-NACT RNASeq was downloaded from GEO. All patients had initial tumor biopsies prior to beginning chemotherapy with six cycles of carboplatin and paclitaxel. The patients subsequently underwent post-chemotherapy surgery, which provided the post-NACT samples. All the samples were obtained from metastatic sites, late recurrence samples n = 11. GSEA on this dataset was performed using the ranked log2 fold-change of late post vs pre chemotherapy samples and Canonical Pathways (c2.cp.v7.4.symbols.gmt).

#### Indentation testing of mouse omental tumors

Mechanical characterization of the specimens was conducted employing an Instron 3342 screwdriver mechanical testing frame (Instron, UK) with a crosshead position resolution of 1 μm. The testing apparatus was equipped with a 10 N load cell (resolution of 0.1 mN) and a flat-ended stainless steel cylindrical probe with a 1 mm diameter for conducting indentation tests. Prior to experimentation, the tissue samples were completely thawed at room temperature. During the entire testing duration, the specimens were fully immersed in phosphate-buffered saline (PBS) at room temperature to maintain full hydration. A pre-load of 1mN was applied to the samples, followed by a deformation to 20% at a constant rate of 1%/s. Subsequently, a displacement-hold period of 600 s was implemented. Data analysis was carried out in accordance with the methodology outlined by Delaine-Smith et al.,^38^ utilizing the mathematical model M2 proposed by,^39^ assuming isotropy in the samples and negligible friction. Throughout the analytical process, a set of well-established parameters were computed, including tangent moduli (TM) within the ranges of 2.5–7.5% and 15–20%, as well as peak modulus (PM), equilibrium modulus (EQM), and percentage of Relaxation. Raw data was processed using Matlab and plotted in GraphPad Prism.

#### Multiplex fluorescent immunohistochemistry (MxIF) staining on mouse HGS2 tumors

For the multiplex immunohistochemistry staining of mouse omental tumors, we used the tissue-cyclic fluorescence (T-CyCIF) methodology^40^ adapted to the Cell DIVE™ (Leica Microsystems) imaging platform.^41^ For the MxIF, serial rounds of conventional immunofluorescence (IF) were facilitated using a fluorophore inactivation step to capture multiple up to 5-plex images on single tissue sections per round for automated downstream registration and assembly into a single high-plex image stack.^41^ 5μm thick FFPE tissue sections were transferred onto SuperFrost Plus™ Adhesion slides (Fisher Scientific, 5067-5579) and baked overnight at 60°C. Tissues then were deparaffinized in xylene and rehydrated in graded washes in ethanol to ddH_2_0. Heat-induced epitope retrieval (HIER) was standardized to a pressure cooker cycle of 116°C for 12 minutes with 1X tris-based antigen unmasking solution (H-3301, Vector) for all antibodies used in the panel. Slides were subsequently incubated for 1h at RT in blocking buffer (10% goat serum/1X Casein/TBST 0.1%), followed by a 60 min incubation at RT in a highly oxidative solution (4.5% H_2_O_2_ and 20mM NaOH in TBS) in the presence of white light. Two LED light sources, emitting 32,000 LUX each, placed on top and on the bottom of the slide tray were used for this photo-bleach / dye Inactivation procedure that aids in tissue auto fluorescence elimination. Upon completion of the bleaching step, the slides were counter stained with Hoechst 33342 (5 μg/mL in TBS) (Thermofisher, H21492) for 10 mins before registering the background” images on the Cell DIVE scanner. After image acquisition, slides were washed and incubated overnight at 4C with the antibody cocktail of the first round. Primary antibodies were either validated by the supplier or against both positive mouse tissue controls and disease-related orthotopic HGS2 mouse model biopsies, using isotype (Table S6C) and secondary-only negative controls in place to gage and subtract non-specific staining. All antibodies used in this study were direct conjugates, apart from F4/80, CD206 and KRT14. F4/80 was conjugated to CoraLite® Plus 750, using the FlexAble CoraLite® Plus 750 Labelling kit (KFA004) following manufacturers’ instructions. Indirect detection was achieved via tissue incubation (30 min, R.T) in primary Ab species-specific secondary Donkey IgG’s conjugated to either AF647 or AF750 dye. Fluorescently tagged secondaries (2’Ab) donkey anti-goat IgG-AF750 (H&L) (ab175745, Abcam) and donkey anti-rabbit IgG-AF647 (Thermofisher, A32795,) were used in conjunction with purified primary antibodies anti-MMR/CD206 Polyclonal (R&D Systems, AF2535) and anti-Cytokeratin 14 clone EPR17350 (Abcam, ab181595), respectively. The full antibody list for the 16-marker panel we used, along with information on dilution used and staining round composition is shown in detail in Table S6D. The next day, slides were once again incubated with Hoechst33342 staining for 5 minutes, washed and then mounted onto ClickWell Assembly cassettes (Leica Microsystems) in 50% glycerol:TBS (vol:vol) imaging solution. Slides were imaged on the CellDIVE platform, using up to four channels plus counterstain with auto fluorescence removal, corrections and stitching, after which the dye inactivation/antibody staining cycling process was reinitiated. For staining rounds 2-6 bleaching was for 45min. Imaging rounds were conducted over a period of 9 days with an intermediate pause of four months for the late PXS-5505 treatment experiment and 13 days for the early PXS-5505 treatment experiment. Slides were stored for long term at 4C for future experiments.

### Quantification and statistical analysis

#### Immunohistochemistry quantification, Haralick feature computation

For standard immunohistochemistry and H&E. tissue sections were scanned with Hamamatsu NanoZoomer S210 Slide-Scanner and the scans analyzed using Definiens® software (Definiens AG, Germany) and QuPath.^42^ QuPath was used to quantify, FN1, VCAN, COL1A1, M3C and LOXL2 immunohistochemistry staining. Difference of Gaussian (DoG) superpixel classification was used to discriminate between adipose and non-adipose tissue. All staining presented were quantified with positive area thresholders. For FN1, VCAN, COL1A1 and M3C staining, a detection area was created out of the positive pixels detected by the thresholder. The Haralick features were computed in the detection area. Results displayed are measurements of non-adipose tissue. As IHC staining of the different mouse experiments occurred on different days, FN1 and M3C staining displayed noticeable batch effect, with the intensity of one or two experiments out of three clearly different from the others. We therefore normalized the Haralick features (computed based on pixel intensity) using the ratio of the medians of the control groups, using the first experiment as the reference.

PCNA-DAB–stained tumour sections were scanned with a Hamamatsu NanoZoomer S210 and quantified in HALO® AI image analysis platform (v3.6.4134), using a custom Multiplex IHC module algorithm. Deconvolution was achieved by colorimetric sampling & thresholding, whilst cell segmentation and tumour classification were optimised through manual sampling and training of the “Nuclei Seg BF v1.0.0 and “DenseNet V2” mask classifiers respectively, with 0.5 and 0.15 entropy endpoints and visual QC. Cytoplasmic segmentation consisted of a 2.5μm radius expansion rule but was not required for positivity mark-up.

#### ECM structure quantification using TWOMBLI

ECM structure was analyzed using TWOMBLI^20^ a plugin for FIJI (www.imagej.net). Representative images of whole slide image sections that were stained with Masson’s trichrome, COL1A1, FN1 and VCAN were extracted under x20 magnification as .tiffs and underwent a color deconvolution step before analysis by TWOMBLI, using the Color deconvolution plugin in FIJI. For Masson’s Trichrome, color deconvolution using the H PAS vector in FIJI, allowed for the isolation of blue collagen from the image, whereas for the remaining stainings, H DAB vector was used to isolate the brown staining that corresponded to Col1A1, FN1 or VCAN. The parameters used for all different stainings for both mouse and human samples are shown in Table S6B. Branch points and endpoints were normalized by dividing by total length; the average fiber thickness was calculated by multiplying high-density matrix with image area and dividing by the total fiber length.

#### Multiplex immunofluorescence image acquisition and quantification

Up to 5-plex immunofluorescent Images were acquired with the Cell DIVE ™ (Leica Microsystems) MxIF platform at 20X. Fully stitched Cell DIVE images were imported, fused, segmented and analyzed using the HALO® (indica Labs) v4.0 software. Tissues were classified to segregate adipose, immune aggregates and “tumor” area (non-adipose, excluding immune aggregates). These classifications were masked as annotations and within each along with within the whole tissue, all markers were quantified with HighPlex FL and Area Quantification FL algorithms. This allowed the identification of phenotypes: cytotoxic T cells (CD8^+^), CD4^+^ cells, B cells (B220+), FoxP3+ cells, TAMs (CD206+ and/or F4/80+ and/or CD163+ and aSMA-), CAFs (aSMA+ and or S100A4+ and CK14- and CK19- and F4/80-). In addition, to quantify the density and distribution of the different cell types regarding the Tumor area, Infiltration Analysis was run for all phenotypes with the whole tissue as Tissue Annotation Layer and using the Tumor annotation as the Interface layer, with a 500um range and 25 bands.

#### Statistical analysis

GraphPad Prim 10 and R v4.1 were used for statistical analyses. Two-sided non-parametric t-test (Mann-Whitney) or unpaired t-test were used to assess differences between conditions. When more than two groups were compared, we used non-parametric one-way ANOVA (Kruskall-Wallis). For the assessment of the effect of two factors (for the spatial analysis) we used a two-way ANOVA. Statistical tests used, n numbers and p values are displayed in the appropriate figures and figure legends. P values < 0.05 were considered statistically significant, with asterisks denoting the level of significance: ∗*p* <= 0.05, ∗∗*p* <= 0.01, ∗∗∗*p* <= 0.001, ∗∗∗∗*p* <= 0.0001.

## Supplemental information

Document S1. Figures S1–S6

Table S1. Gene cluster membership, annotation, and gene ontology biological pathways enrichment, related to Figure 1

Table S2. GSVA enrichment scores using the Bagaev et al.’s 14 functional gene expression signatures, related to Figure 1

Table S3. Overlapping canonical pathways in 60577 treated vs. control TP1 or TP2 and the Adzibolosu et al.’s dataset (GSE227100), related to Figure 1

Table S4. Normalized gene expression and differential gene expression statistics of malignant cell line RNA-seq, related to Figures 4 and S4

Table S5. Spearman’s correlation coefficients for ECM genes with structure index in 60577, but not in HGS2, related to Figures 4 and S4

Table S6. Antibodies and conditions for immunohistochemistry, Cell DIVE, and ECM TWOMBLI analysis

## References

[1] Closset L., Gultekin O., Salehi S., Sarhan D., Lehti K., Gonzalez-Molina J. (2023). The extracellular matrix - immune microenvironment crosstalk in cancer therapy: Challenges and opportunities. Matrix Biol..

[2] Winkler J., Abisoye-Ogunniyan A., Metcalf K.J., Werb Z. (2020). Concepts of extracellular matrix remodelling in tumour progression and metastasis. Nat. Commun..

[3] Gonzalez-Molina J., Moyano-Galceran L., Single A., Gultekin O., Alsalhi S., Lehti K. (2022). Chemotherapy as a regulator of extracellular matrix-cell communication: Implications in therapy resistance. Semin. Cancer Biol..

[4] Pietilä E.A., Gonzalez-Molina J., Moyano-Galceran L., Jamalzadeh S., Zhang K., Lehtinen L., Turunen S.P., Martins T.A., Gultekin O., Lamminen T. (2021). Co-evolution of matrisome and adaptive adhesion dynamics drives ovarian cancer chemoresistance. Nat. Commun..

[5] Pearce O.M.T., Delaine-Smith R.M., Maniati E., Nichols S., Wang J., Böhm S., Rajeeve V., Ullah D., Chakravarty P., Jones R.R. (2018). Deconstruction of a Metastatic Tumor Microenvironment Reveals a Common Matrix Response in Human Cancers. Cancer Discov..

[6] Webb P.M., Jordan S.J. (2024). Global epidemiology of epithelial ovarian cancer. Nat. Rev. Clin. Oncol..

[7] Mahmood R.D., Morgan R.D., Edmondson R.J., Clamp A.R., Jayson G.C. (2020). First-Line Management of Advanced High-Grade Serous Ovarian Cancer. Curr. Oncol. Rep..

[8] Bohm S., Montfort A., Pearce O.M., Topping J., Chakravarty P., Everitt G.L., Clear A., McDermott J.R., Ennis D., Dowe T. (2016). Neoadjuvant Chemotherapy Modulates the Immune Microenvironment in Metastases of Tubo-Ovarian High-Grade Serous Carcinoma. Clin. Cancer Res..

[9] Berlato C., Khan M.N., Schioppa T., Thompson R., Maniati E., Montfort A., Jangani M., Canosa M., Kulbe H., Hagemann U.B. (2017). A CCR4 antagonist reverses the tumor-promoting microenvironment of renal cancer. J. Clin. Investig..

[10] Heath O., Berlato C., Maniati E., Lakhani A., Pegrum C., Kotantaki P., Elorbany S., Böhm S., Barry S.T., Annibaldi A. (2021). Chemotherapy induces tumor-associated macrophages that aid adaptive immune responses in ovarian cancer. Cancer Immunol. Res..

[11] Elorbany S., Berlato C., Carnevalli L.S., Maniati E., Barry S.T., Wang J., Manchanda R., Kzhyshkowska J., Balkwill F. (2024). Immunotherapy that improves response to chemotherapy in high-grade serous ovarian cancer. Nat. Commun..

[12] Galluzzi L., Humeau J., Buqué A., Zitvogel L., Kroemer G. (2020). Immunostimulation with chemotherapy in the era of immune checkpoint inhibitors. Nat. Rev. Clin. Oncol..

[13] Maniati E., Berlato C., Gopinathan G., Heath O., Kotantaki P., Lakhani A., McDermott J., Pegrum C., Delaine-Smith R.M., Pearce O.M.T. (2020). Mouse Ovarian Cancer Models Recapitulate the Human Tumor Microenvironment and Patient Response to Treatment. Cell Rep..

[14] Bagaev A., Kotlov N., Nomie K., Svekolkin V., Gafurov A., Isaeva O., Osokin N., Kozlov I., Frenkel F., Gancharova O. (2021). Conserved pan-cancer microenvironment subtypes predict response to immunotherapy. Cancer Cell.

[15] Adzibolosu N., Alvero A.B., Ali-Fehmi R., Gogoi R., Corey L., Tedja R., Chehade H., Gogoi V., Morris R., Anderson M. (2023). Immunological modifications following chemotherapy are associated with delayed recurrence of ovarian cancer. Front. Immunol..

[16] Wouters M.C.A., Nelson B.H. (2018). Prognostic Significance of Tumor-Infiltrating B Cells and Plasma Cells in Human Cancer. Clin. Cancer Res..

[17] Nielsen J.S., Sahota R.A., Milne K., Kost S.E., Nesslinger N.J., Watson P.H., Nelson B.H. (2012). CD20+ tumor-infiltrating lymphocytes have an atypical CD27- memory phenotype and together with CD8+ T cells promote favorable prognosis in ovarian cancer. Clin. Cancer Res..

[18] Milne K., Köbel M., Kalloger S.E., Barnes R.O., Gao D., Gilks C.B., Watson P.H., Nelson B.H. (2009). Systematic analysis of immune infiltrates in high-grade serous ovarian cancer reveals CD20, FoxP3 and TIA-1 as positive prognostic factors. PLoS One.

[19] Santoiemma P.P., Reyes C., Wang L.P., McLane M.W., Feldman M.D., Tanyi J.L., Powell D.J. (2016). Systematic evaluation of multiple immune markers reveals prognostic factors in ovarian cancer. Gynecol. Oncol..

[20] Wershof E., Park D., Barry D.J., Jenkins R.P., Rullan A., Wilkins A., Schlegelmilch K., Roxanis I., Anderson K.I., Bates P.A., Sahai E. (2021). A FIJI macro for quantifying pattern in extracellular matrix. Life Sci. Alliance.

[21] Devlin M.J., Miller R., Laforets F., Kotantaki P., Garsed D.W., Kristeleit R., Bowtell D.D., McDermott J., Maniati E., Balkwill F.R. (2022). The Tumor Microenvironment of Clear-Cell Ovarian Cancer. Cancer Immunol. Res..

[22] Chitty J.L., Setargew Y.F.I., Cox T.R. (2019). Targeting the lysyl oxidases in tumour desmoplasia. Biochem. Soc. Trans..

[23] Johnston K.A., Lopez K.M. (2018). Lysyl oxidase in cancer inhibition and metastasis. Cancer Lett..

[24] Maller O., Drain A.P., Barrett A.S., Borgquist S., Ruffell B., Zakharevich I., Pham T.T., Gruosso T., Kuasne H., Lakins J.N. (2021). Tumour-associated macrophages drive stromal cell-dependent collagen crosslinking and stiffening to promote breast cancer aggression. Nat. Mater..

[25] Patch A.M., Christie E.L., Etemadmoghadam D., Garsed D.W., George J., Fereday S., Nones K., Cowin P., Alsop K., Bailey P.J. (2015). Whole-genome characterization of chemoresistant ovarian cancer. Nature.

[26] Chitty J.L., Yam M., Perryman L., Parker A.L., Skhinas J.N., Setargew Y.F.I., Mok E.T.Y., Tran E., Grant R.D., Latham S.L. (2023). A first-in-class pan-lysyl oxidase inhibitor impairs stromal remodeling and enhances gemcitabine response and survival in pancreatic cancer. Nat. Cancer.

[27] Mierke C.T. (2021). Viscoelasticity Acts as a Marker for Tumor Extracellular Matrix Characteristics. Front. Cell Dev. Biol..

[28] Chirivì M., Maiullari F., Milan M., Presutti D., Cordiglieri C., Crosti M., Sarnicola M.L., Soluri A., Volpi M., Święszkowski W. (2021). Tumor Extracellular Matrix Stiffness Promptly Modulates the Phenotype and Gene Expression of Infiltrating T Lymphocytes. Int. J. Mol. Sci..

[29] Du H., Bartleson J.M., Butenko S., Alonso V., Liu W.F., Winer D.A., Butte M.J. (2023). Tuning immunity through tissue mechanotransduction. Nat. Rev. Immunol..

[30] Burchard P.R., Ruffolo L.I., Ullman N.A., Dale B.S., Dave Y.A., Hilty B.K., Ye J., Georger M., Jewell R., Miller C. (2024). Pan-lysyl oxidase inhibition disrupts fibroinflammatory tumor stroma, rendering cholangiocarcinoma susceptible to chemotherapy. Hepatol. Commun..

[31] Nicolas-Boluda A., Vaquero J., Vimeux L., Guilbert T., Barrin S., Kantari-Mimoun C., Ponzo M., Renault G., Deptula P., Pogoda K. (2021). Tumor stiffening reversion through collagen crosslinking inhibition improves T cell migration and anti-PD-1 treatment. eLife.

[32] Kagan H.M., Sullivan K.A., Olsson T.A., Cronlund A.L. (1979). Purification and properties of four species of lysyl oxidase from bovine aorta. Biochem. J..

[33] Palamakumbura A.H., Trackman P.C. (2002). A fluorometric assay for detection of lysyl oxidase enzyme activity in biological samples. Anal. Biochem..

[34] Dobin A., Gingeras T.R. (2015). Mapping RNA-seq Reads with STAR. Curr. Protoc. Bioinformatics.

[35] Li B., Dewey C.N. (2011). RSEM: accurate transcript quantification from RNA-Seq data with or without a reference genome. BMC Bioinf..

[36] Ritchie M.E., Phipson B., Wu D., Hu Y., Law C.W., Shi W., Smyth G.K. (2015). limma powers differential expression analyses for RNA-sequencing and microarray studies. Nucleic Acids Res..

[37] Hanzelmann S., Castelo R., Guinney J. (2013). GSVA: gene set variation analysis for microarray and RNA-seq data. BMC Bioinf..

[38] Delaine-Smith R.M., Burney S., Balkwill F.R., Knight M.M. (2016). Experimental validation of a flat punch indentation methodology calibrated against unconfined compression tests for determination of soft tissue biomechanics. J. Mech. Behav. Biomed. Mater..

[39] Oliver W.C., Pharr G.M. (1992). An improved technique for determining hardness and elastic modulus using load and displacement sensing indentation experiments. J. Mater. Res..

[40] Lin J.R., Izar B., Wang S., Yapp C., Mei S., Shah P.M., Santagata S., Sorger P.K. (2018). Highly multiplexed immunofluorescence imaging of human tissues and tumors using t-CyCIF and conventional optical microscopes. eLife.

[41] Gerdes M.J., Sevinsky C.J., Sood A., Adak S., Bello M.O., Bordwell A., Can A., Corwin A., Dinn S., Filkins R.J. (2013). Highly multiplexed single-cell analysis of formalin-fixed, paraffin-embedded cancer tissue. Proc. Natl. Acad. Sci. USA.

[42] Bankhead P., Loughrey M.B., Fernández J.A., Dombrowski Y., McArt D.G., Dunne P.D., McQuaid S., Gray R.T., Murray L.J., Coleman H.G. (2017). QuPath: Open source software for digital pathology image analysis. Sci. Rep..

