# Supplementary material for: Matrix structure and microenvironment dynamics correlate with chemotherapy response in ovarian cancer
Source: iScience. 2026 Feb 9;29(3):114858. doi: 10.1016/j.isci.2026.114858 (PMC13080397; doi:10.1016/j.isci.2026.114858)
Supplement: Document S1. Figures S1–S6 [file mmc1.pdf]

iScience, Volume *29*

Supplemental information

Matrix structure and microenvironment dynamics correlate with chemotherapy response in ovarian cancer

Florian Laforêts, Panoraia Kotantaki, Samar Elorbany, Joseph Hartlebury, Joash D. Joy, Beatrice Malacrida, Rachel C. Bryan-Ravenscroft, Chiara Berlato, Erica Di Federico, John F. Marshall, Ranjit Manchanda, Wolfgang Jarolimek, Lara Perryman, Eleni Maniati, and Frances R. Balkwill

Figure S1

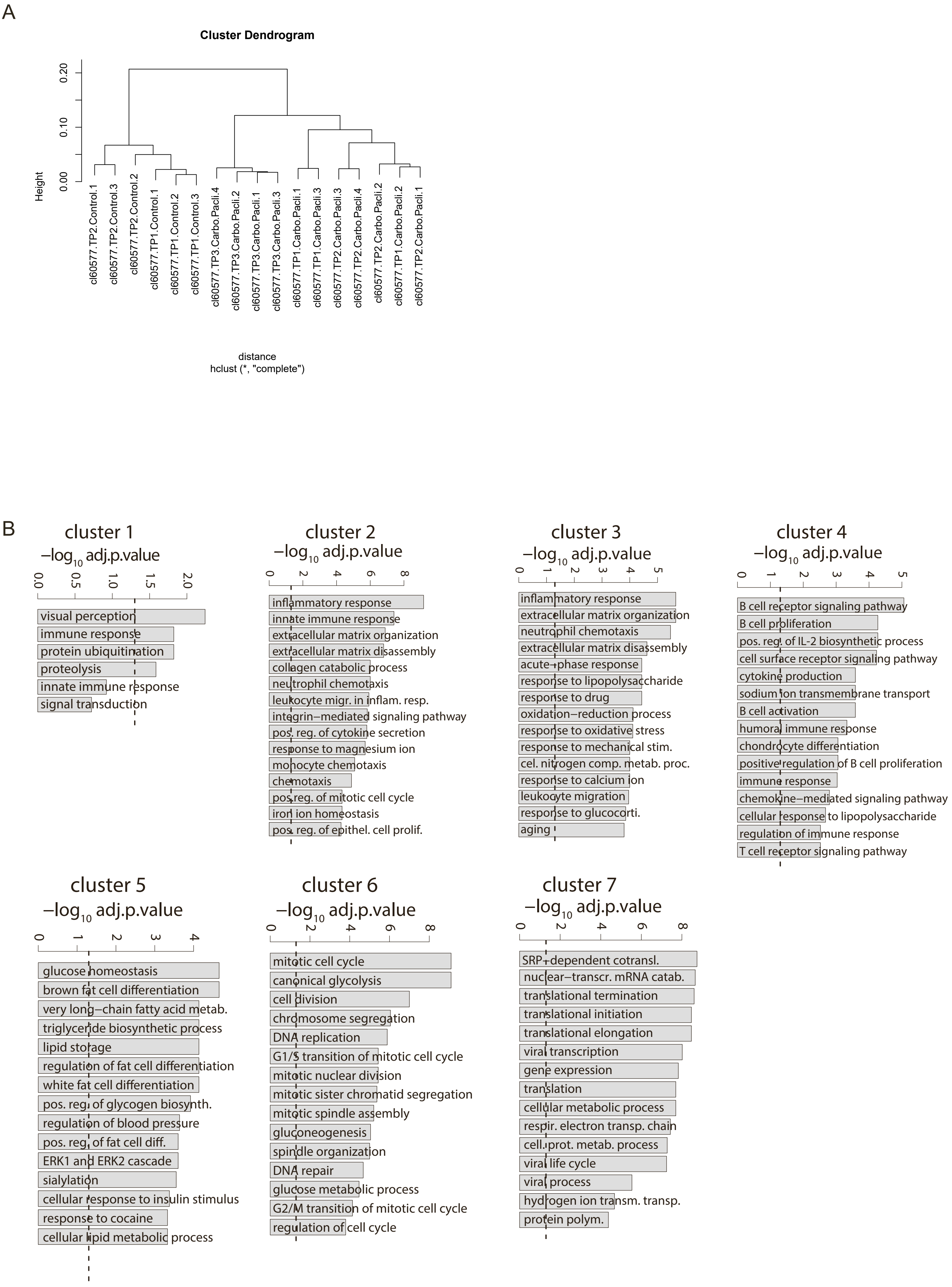
A
Cluster Dendrogram
Height
distance
hclust (*, "complete")
cl60577.TP2.Control.1
cl60577.TP2.Control.3
cl60577.TP2.Control.2
cl60577.TP1.Control.1
cl60577.TP1.Control.2
cl60577.TP1.Control.3
cl60577.TP3.Carbo.Pacli.4
cl60577.TP3.Carbo.Pacli.2
cl60577.TP3.Carbo.Pacli.1
cl60577.TP3.Carbo.Pacli.3
cl60577.TP1.Carbo.Pacli.1
cl60577.TP1.Carbo.Pacli.3
cl60577.TP2.Carbo.Pacli.3
cl60577.TP2.Carbo.Pacli.4
cl60577.TP2.Carbo.Pacli.2
cl60577.TP1.Carbo.Pacli.2
cl60577.TP2.Carbo.Pacli.1
B
cluster 1
−log10 adj.p.value
visual perception
immune response
protein ubiquitination
proteolysis
innate immune response
signal transduction
cluster 2
−log10 adj.p.value
inflammatory response
innate immune response
extracellular matrix organization
extracellular matrix disassembly
collagen catabolic process
neutrophil chemotaxis
leukocyte migr. in inflam. resp.
integrin−mediated signaling pathway
pos. reg. of cytokine secretion
response to magnesium ion
monocyte chemotaxis
chemotaxis
pos.reg. of mitotic cell cycle
iron ion homeostasis
pos. reg. of epithel. cell prolif.
cluster 3
−log10 adj.p.value
inflammatory response
extracellular matrix organization
neutrophil chemotaxis
extracellular matrix disassembly
acute−phase response
response to lipopolysaccharide
response to drug
oxidation−reduction process
response to oxidative stress
response to mechanical stim.
cel. nitrogen comp. metab. proc.
response to calcium ion
leukocyte migration
response to glucocorti.
aging
cluster 4
−log10 adj.p.value
B cell receptor signaling pathway
B cell proliferation
pos. reg. of IL-2 biosynthetic process
cell surface receptor signaling pathway
cytokine production
sodium ion transmembrane transport
B cell activation
humoral immune response
chondrocyte differentiation
positive regulation of B cell proliferation
immune response
chemokine−mediated signaling pathway
cellular response to lipopolysaccharide
regulation of immune response
T cell receptor signaling pathway
cluster 5
−log10 adj.p.value
glucose homeostasis
brown fat cell differentiation
very long−chain fatty acid metab.
triglyceride biosynthetic process
lipid storage
regulation of fat cell differentiation
white fat cell differentiation
pos. reg. of glycogen biosynth.
regulation of blood pressure
pos. reg. of fat cell diff.
ERK1 and ERK2 cascade
sialylation
cellular response to insulin stimulus
response to cocaine
cellular lipid metabolic process
cluster 6
−log10 adj.p.value
mitotic cell cycle
canonical glycolysis
cell division
chromosome segregation
DNA replication
G1/S transition of mitotic cell cycle
mitotic nuclear division
mitotic sister chromatid segregation
mitotic spindle assembly
gluconeogenesis
spindle organization
DNA repair
glucose metabolic process
G2/M transition of mitotic cell cycle
regulation of cell cycle
cluster 7
−log10 adj.p.value
SRP−dependent cotransl.
nuclear−transcr. mRNA catab.
translational termination
translational initiation
translational elongation
viral transcription
gene expression
translation
cellular metabolic process
respir. electron transp. chain
cell. prot. metab. process
viral life cycle
viral process
hydrogen ion transm. transp.
protein polym.

Figure S1 related to Figure 1: Supplemental bulk RNA seq data for 60577. A. Hierarchical cluster analysis based on Pearson's correlation matrix of all genes in 60577 tumors and the complete clustering method. **B**. Barplots illustrating the top significantly enriched Gene Ontology Biological Processes of the genes in the clusters 1 to 7 (broken line indicates adjp = 0.05)

Figure S2

A

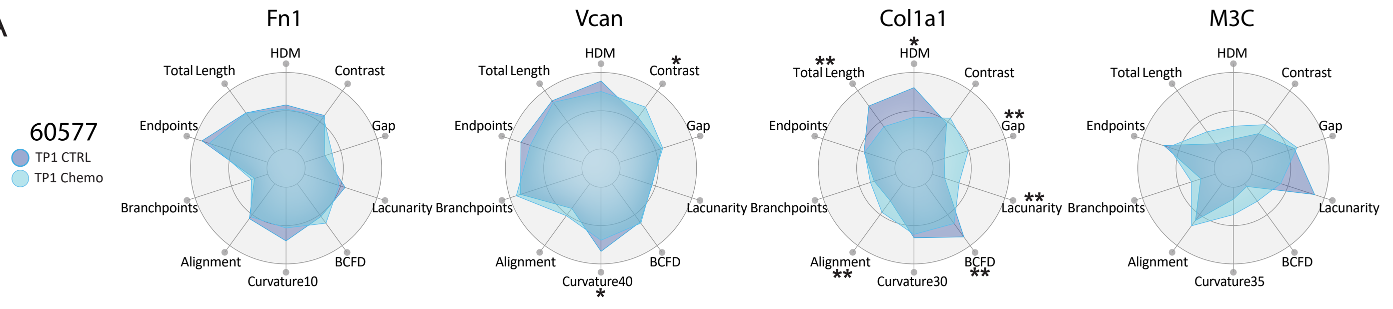

B

PFS post only

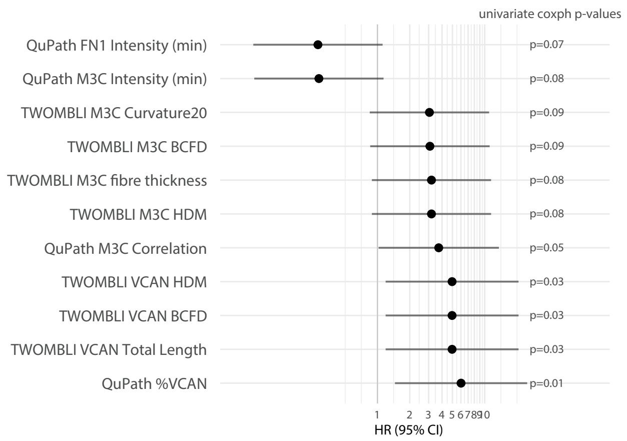

Figure S2 related to Figure 2: Effect of chemotherapy on ECM structure at TP1 in 60577 and correlation of PFS with ECM structure in human

A. Matrix patterns for FN1, VCAN, COL1A1 and Masson's Trichrome (M3C) for 60577 TP1, as they were assessed by TWOMBLI and Haralick feature analysis and visualized in the radar plots. The same selection of TWOMBLI and Haralick metrics as used for TP2 is depicted. dark and light blue show the ECM pattern for control and treated TP1 tumors, respectively. *p<=0.05, **p<= 0.01, Mann-Whitney test. **B.** Univariate Cox Proportional Hazards Regression Analysis on progression-free survival using TWOMBLI and QuPath image analysis IHC metrics on post NACT samples to split patient groups (human). High and low groups were determined by median values n_{high} = 7, n_{low} = 8. Hazard ratios greater than zero denote increased risk in high group; Hazard ratios lower than zero denote lower risk in high group.

Figure S3

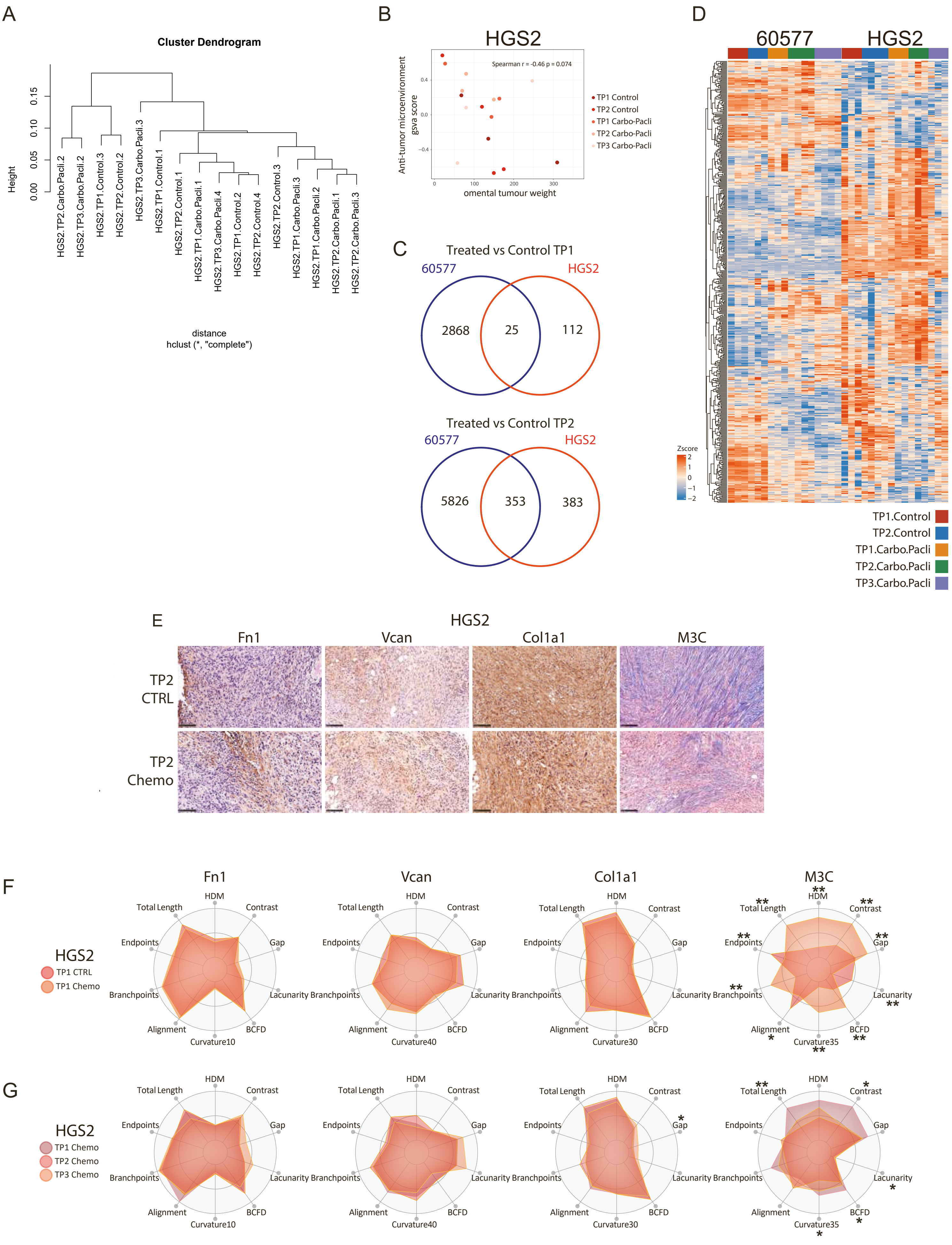
A
Cluster Dendrogram
Height
0.00
0.05
0.10
0.15
HGS2.TP2.Carbo.Pacli.2
HGS2.TP3.Carbo.Pacli.2
HGS2.TP1.Control.3
HGS2.TP2.Control.2
HGS2.TP3.Carbo.Pacli.3
HGS2.TP1.Control.1
HGS2.TP2.Control.1
HGS2.TP1.Carbo.Pacli.1
HGS2.TP3.Carbo.Pacli.4
HGS2.TP1.Control.2
HGS2.TP2.Control.4
HGS2.TP2.Control.3
HGS2.TP1.Carbo.Pacli.3
HGS2.TP1.Carbo.Pacli.2
HGS2.TP2.Carbo.Pacli.1
HGS2.TP2.Carbo.Pacli.3
distance
hclust (*, "complete")
B
HGS2
Spearman r = -0.46 p = 0.074
Anti-tumor microenvironment
gsva score
omental tumour weight
TP1 Control
TP2 Control
TP1 Carbo-Pacli
TP2 Carbo-Pacli
TP3 Carbo-Pacli
C
Treated vs Control TP1
60577
HGS2
2868
25
112
Treated vs Control TP2
60577
HGS2
5826
353
383
D
60577
HGS2
Zscore
TP1.Control
TP2.Control
TP1.Carbo.Pacli
TP2.Carbo.Pacli
TP3.Carbo.Pacli
E
HGS2
Fn1
Vcan
Col1a1
M3C
TP2 CTRL
TP2 Chemo
F
HGS2
TP1 CTRL
TP1 Chemo
Fn1
Vcan
Col1a1
M3C
HDM
Contrast
Gap
Lacunarity
BCFD
Curvature10
Curvature40
Curvature30
Curvature35
Alignment
Branchpoints
Endpoints
Total Length
G
HGS2
TP1 Chemo
TP2 Chemo
TP3 Chemo

Figure S3 related to Figure 3: Effect of chemotherapy on the transcriptome and ECM in HGS2

A. Hierarchical cluster analysis based on Pearson's correlation matrix of all genes in HGS2 tumors and the complete clustering method. **B.** Correlation scatter plot of the anti-tumor microenvironment scores with omental tumor weights in 60577. Each dot represents an individual mouse tumor**.** Points that correspond to different experimental groups are illustrated in different colors. **C.** Venn diagrams illustrating overlap of differentially expressed genes (unadjusted p <= 0.01) in treated versus control at TP1 and TP2 for 60577 and HGS2. **D**. Heatmap of HGS2 unique genes in treated versus control at TP1 and/or TP2. **E.** Representative images of immunohistochemistry for fibronectin (FN1), versican (VCAN), Collagen 1A1 (COL1A1) immunohistochemistry and Masson's Trichrome (M3C) histochemistry of HGS2 tumors taken at TP2. Scale bars, 100μm. **F**. ECM patterns for FN1, VCAN, COL1A1 and Masson's Trichrome (M3C) for HGS2 tumors at TP1, as they were assessed by TWOMBLI and Haralick feature analysis and visualized as radar plots. A selection of TWOMBLI metrics along with Haralick contrast is depicted. The average of structural and textural metrics was calculated and the minimum and maximum range for each metric was computed across all categories. Values were normalized and scaled to the maximum range. Red and orange show the ECM pattern for control and treated TP1 tumors, respectively. **G**. ECM patterns for treated TP1, TP2 and TP3 tumors in Red, salmon and orange, respectively. HDM: High Density Matrix, Gap: Mean Gap Area, BCFD: Box Counting Fractal Dimension, Curvature10 to 40: Curvature metric assessed at windows of 10 to 40 pixels, Branchpoints: normalized branchpoints, Endpoints: normalized endpoints, Fiber Length: Average Fiber length**.** *p<=0.05, **p<= 0.01, Mann-Whitney test.

Figure S4

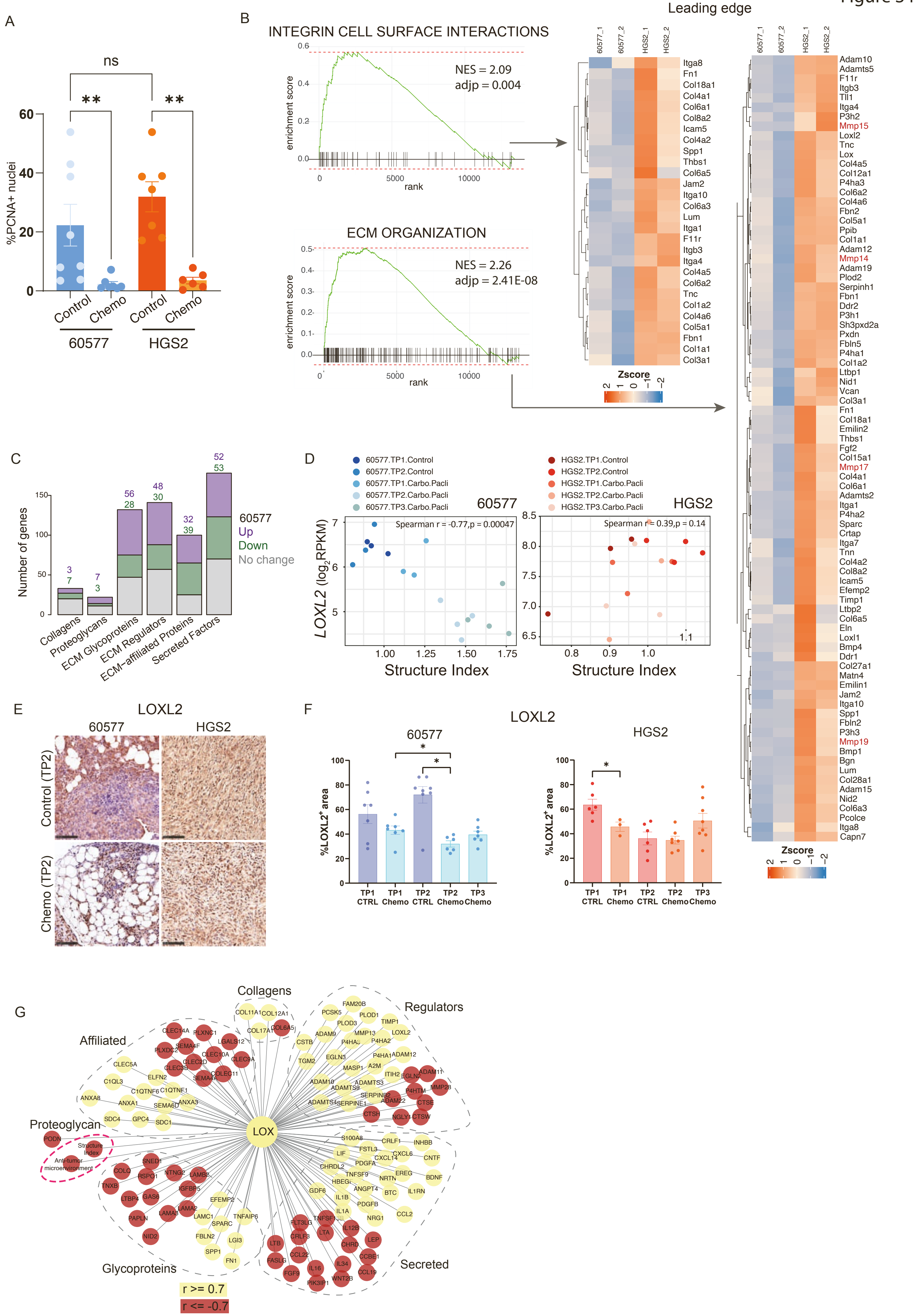

A
ns
%PCNA+ nuclei
Control
Chemo
Control
Chemo
60577
HGS2
B
INTEGRIN CELL SURFACE INTERACTIONS
enrichment score
rank
NES = 2.09
adjp = 0.004
ECM ORGANIZATION
NES = 2.26
adjp = 2.41E-08
Leading edge
Zscore
C
Number of genes
60577
Up
Down
No change
Collagens
Proteoglycans
ECM Glycoproteins
ECM Regulators
ECM-affiliated Proteins
Secreted Factors
D
60577.TP1.Control
60577.TP2.Control
60577.TP1.Carbo.Pacli
60577.TP2.Carbo.Pacli
60577.TP3.Carbo.Pacli
HGS2.TP1.Control
HGS2.TP2.Control
HGS2.TP1.Carbo.Pacli
HGS2.TP2.Carbo.Pacli
HGS2.TP3.Carbo.Pacli
Spearman r = -0.77,p = 0.00047
Spearman r = 0.39,p = 0.14
LOXL2 (log2RPKM)
Structure Index
E
LOXL2
Control (TP2)
Chemo (TP2)
F
%LOXL2+ area
TP1 CTRL
TP1 Chemo
TP2 CTRL
TP2 Chemo
TP3 Chemo
G
Collagens
Regulators
Affiliated
Proteoglycan
Glycoproteins
Secreted
LOX
Structure Index
Anti-tumor microenvironment
r >= 0.7
r <= -0.7

Figure S4 related to Figure 4. LOX family expression and correlation network

A. Quantification of PCNA+ nuclei on IHC images of PCNA staining in control or chemo treated tumours at TP2. Data are shown as mean ± sem , **p<= 0.01, Mann-Whitney test. $60577_{Control}$ n=7, 60577_{Chemo} n=6, $HGS2_{Control}$ n=7, $HGS2_{Chemo}$ n=6. **B**. RNASeq was performed on the 60577 and HGS2 cell lines, grown in 2D monocultures. Enrichment plots of GSEA pre-ranked analysis using the ranked t-statistic of HGS2 versus 60577 cell lines illustrate significantly higher enrichment of Reactome integrin cell surface interactions and ECM organization pathways in HGS2 compared to 60577. Heatmaps illustrate normalized expression (log_2RPKM) of the leading edge genes from the two enrichment plots are included. **C**. Stacked barplot illustrating the number of matrisome genes per matrisome category that had a positive or negative association with structure index in 60577 but not in HGS2 (Spearman's r >= |0.5| and p <= 0.05) **D.** Correlation scatter plots illustrating *Loxl2* correlation with structure index in both mouse models, each dot represents an individual mouse. **E**. Immunohistochemical staining and **F.** Quantification for *LOXL2* in control and chemotherapy treated tumors from 60577 (blue) and HGS2 models (orange). % positive area in the tumor plus stromal area (excluding fat) was quantified in both cases. Data are shown as mean ± sem, each dot represents a mouse, p-values correspond to Kruskal-Wallis test and Mann-Whitney for individual time points * p<0.05. A representative image from TP2 is shown from each tumor, scale bar: 100um.

G. Correlation network constructed using the matrisome genes with a positive (pearson's r>=0.7, p <= 0.05) or negative correlation (pearson's r>=0.7, p <= 0.05) with LOX. Anti-TME score and structure index circled in fuchsia.

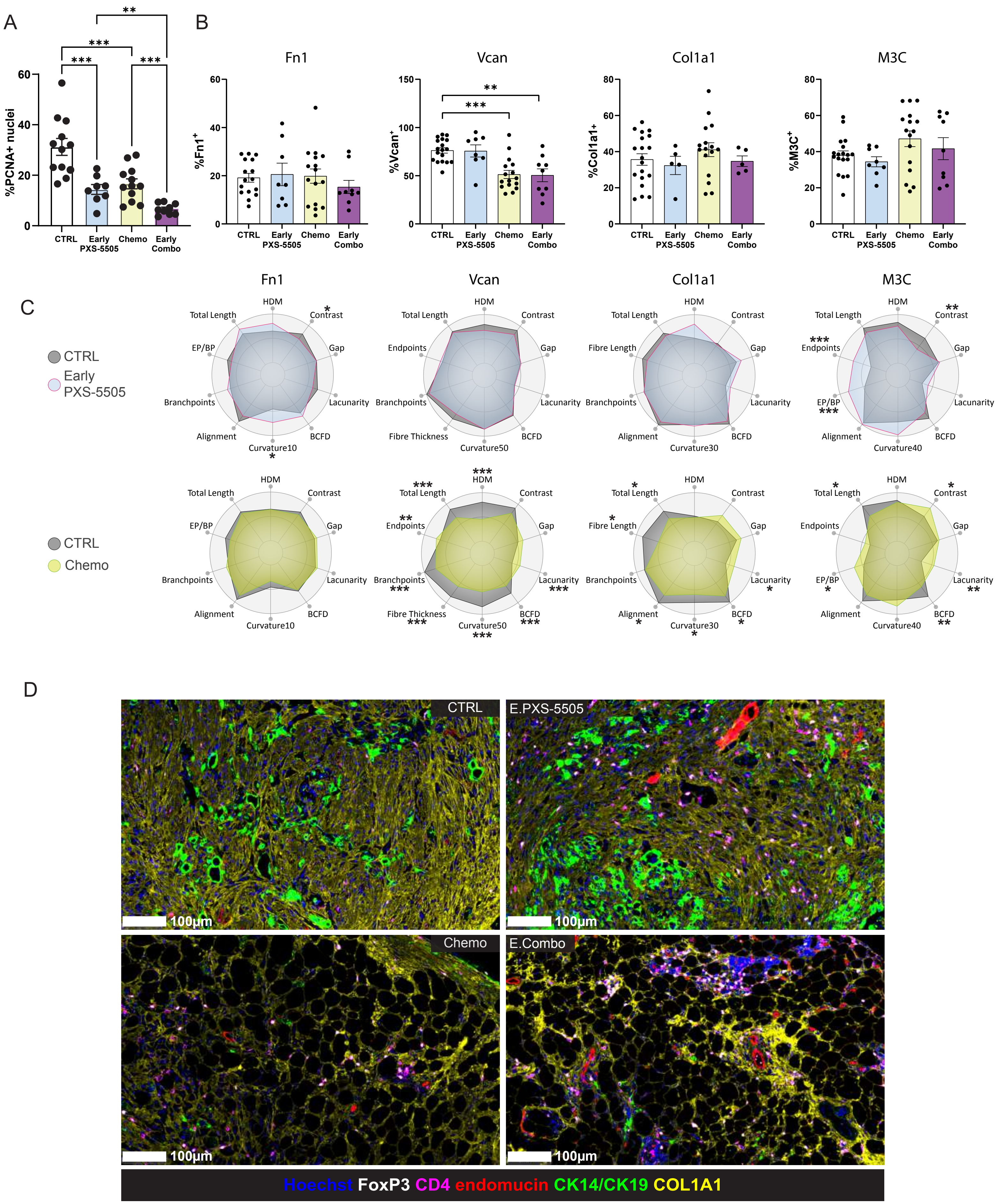
A
B
C
D
%PCNA+ nuclei
CTRL
Early PXS-5505
Chemo
Early Combo
Fn1
Vcan
Col1a1
M3C
%Fn1+
%Vcan+
%Col1a1+
%M3C+
HDM
Contrast
Gap
Lacunarity
BCFD
Curvature10
Curvature50
Curvature30
Curvature40
Alignment
Branchpoints
EP/BP
Endpoints
Total Length
Fibre Thickness
Fibre Length
CTRL
Early PXS-5505
CTRL
Chemo
CTRL
E.PXS-5505
Chemo
E.Combo
100µm
Hoechst FoxP3 CD4 endomucin CK14/CK19 COL1A1

Figure S5 related to Figure 5. Effect of chemotherapy and prolonged PXS-5505 on ECM structure and TME composition in HGS2

A. Fluorescent immunohistochemistry staining for PCNA in HGS2 TP2 tumors treated with chemo +/- early PXS-5505. Representative images for PCNA staining of control or treated tumours at TP2 along with quantification of PCNA+ nuclei. Data are shown as mean ± sem, **p<= 0.01, ***p<= 0.001, Mann-Whitney test. Control n=12, Early PXS-5505 n=8, Chemo n = 12, Early Combo n = 9. **B**. Quantification of % FN1+, VCAN+, COL1A1+ and M3C+ area for HGS2 biopsies treated with early PXS-5505 in the presence or in the absence of chemotherapy**.** p-values correspond to two-tailed Mann-Whitney U test. Control n=16-18, Chemo n=16, Early PXS-5505 n=8, Early combo n=9. Each dot represents an individual mouse. Data are shown as mean ± sem, **p<= 0.01, ***p<= 0.001, Mann-Whitney test. **C.** Structural and textural modifications of ECM induced by early treatment with PXS-5505 and chemotherapy in HGS2 tumors at TP2. Radar plots computed as previously, show selected metrics for FN1, VCAN, COL1A1 and M3C fibers. The scaled average for each group is depicted. Control in grey, early PXS-5505 in light blue, Chemo group in yellow. P-values correspond to two-tailed Mann-Whitney U test, *p<=0.05, **p<= 0.01, ***p<= 0.001. Data pooled from two individual experiments are shown, along with controls and chemo treated samples from a third experiment. Control n=16-18, Chemo n=16, Early PXS-5505 n=8. **D.** A representative composite image from each treatment group of HGS2 at TP2 is shown with CD4 (fuschia), FoxP3 (white), endomucin (red), cancer cells (CK14/CK19, green) and COL1A1 (yellow). Hoechst 33342 in blue. E.PXS-5505: early PXS-5505, E.Combo: Early Combo. Scale bar is 100um.

Figure S6

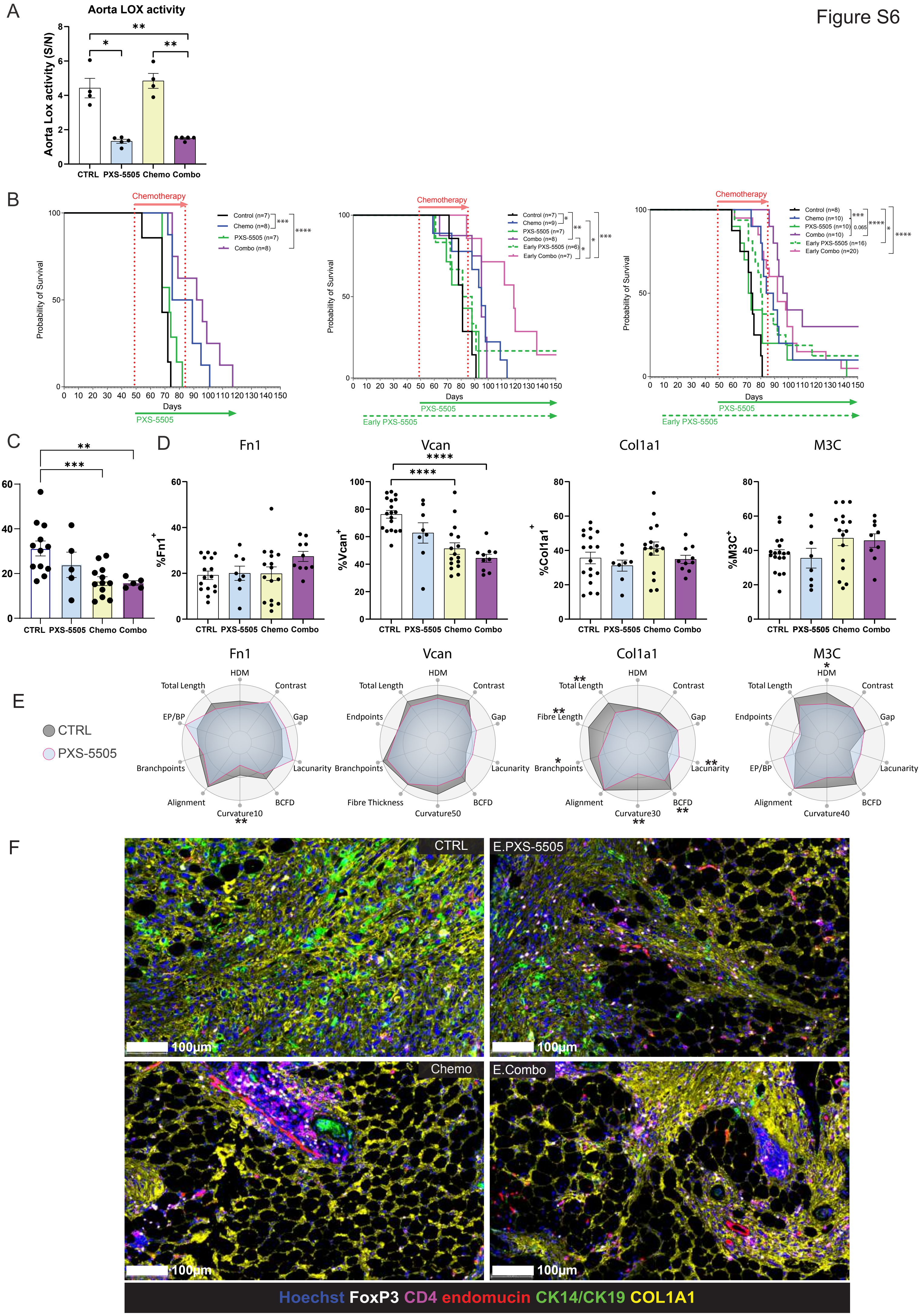

A
Aorta LOX activity
Aorta Lox activity (S/N)
CTRL PXS-5505 Chemo Combo
B
Chemotherapy
Probability of Survival
Days
PXS-5505
Early PXS-5505
Control (n=7)
Chemo (n=8)
PXS-5505 (n=7)
Combo (n=8)
Chemo (n=9)
Early PXS-5505 (n=6)
Early Combo (n=7)
Control (n=8)
Chemo (n=10)
PXS-5505 (n=10)
Combo (n=10)
Early PXS-5505 (n=16)
Early Combo (n=20)
0.065
C
%PCNA+ nuclei
D
Fn1
Vcan
Col1a1
M3C
E
CTRL
PXS-5505
HDM
Contrast
Gap
Lacunarity
BCFD
Curvature10
Alignment
Branchpoints
EP/BP
Total Length
Endpoints
Fibre Thickness
Curvature50
Fibre Length
Curvature30
Curvature40
F
CTRL
E.PXS-5505
Chemo
E.Combo
100μm
Hoechst FoxP3 CD4 endomucin CK14/CK19 COL1A1

Figure S6 related to Figure 6. Effect of chemotherapy and concomitant PXS-5505 treatment on ECM structure and TME composition in HGS2

A. Lysyl oxidase family activity measured in snap frozen aortas determined by fluorometric activity assay. Number of mice were for control *n* = 4, late PXS-5505 n=5, chemo n=4 and late combo n=5. Data presented as mean ± sem, *p<=0.05, **p<= 0.01. Two-tailed *p* value determined by unpaired, nonparametric *t*-test with a Mann–Whitney *U*-test correction. **B.** Survival curves for the three individual *in vivo* experiments described in Fig 5B and Fig 6B, using early and late treatment with PXS-5505 and six cycles of chemotherapy (carboplatin 20 mg/kg, paclitaxel 10mg/kg, once per week). Median survival for control is 68, 81 and 73.5 days, for the 1st, 2nd and 3rd experiment, for chemo 82, 95 and 86.5, for late PXS-5505, 73, 81, and 72, for late combo, 94, 95 and 97, respectively. For early PXS-5505, median survival was 84 and 80 days for the 2nd and 3rd experiment and for early combo 119 and 92 days, respectively. The log rank p value is depicted on the survival curves, *p<=0.05, **p<= 0.01, ***p<= 0.001, ****p<= 0.0001. The start of the treatment is indicated by the red arrow for chemotherapy, green arrow is for PXS-5505. Number of mice enrolled in each arm is shown in parentheses. **C.** Fluorescent immunohistochemistry staining for PCNA in HGS2 TP2 tumors treated with chemo +/- PXS-5505. Representative images for PCNA staining of control or treated tumors at TP2 along with quantification of PCNA+ nuclei. Data are shown as mean ± sem, **p<= 0.01, ***p<= 0.001, Mann-Whitney test. Control n=12, PXS-5505 n=5, Chemo n = 12, Combo n = 5. **D**. Quantification of % FN1+, VCAN+, COL1A1+ and M3C+ area in HGS2 biopsies treated or not with PXS-5505 and in the presence or in the absence of chemotherapy. p-values correspond to two-tailed Mann-Whitney U test, ****p<= 0.001. Control n=16-18, chemo n=16, late PXS-5505 n=8, late combo n=10.

Each dot represents an individual mouse. **E.** Structural and textural modifications of ECM induced by late treatment with PXS-5505 in HGS2 tumors at TP2. Radar plots show selected metrics for FN1, VCAN, COL1A1 and M3C fibers. Control is shown in grey, late PXS-5505 in light blue. P-values correspond to two-tailed Mann-Whitney U test, *p<=0.05, **p<= 0.01. **F.** Representative composite image from each treatment group HGS2 at TP2 is shown with CD4 (fuchsia), FoxP3 (white), endomucin (red), cancer cells (CK14/CK19, green) and COL1A1 (yellow). Hoechst 33342 in blue. Scale bar is 100um.
